# Universal momentum-to-real-space mapping of topological singularities

**DOI:** 10.1038/s41467-020-15374-x

**Published:** 2020-03-27

**Authors:** Xiuying Liu, Shiqi Xia, Ema Jajtić, Daohong Song, Denghui Li, Liqin Tang, Daniel Leykam, Jingjun Xu, Hrvoje Buljan, Zhigang Chen

**Affiliations:** 1 The MOE Key Laboratory of Weak-Light Nonlinear Photonics, TEDA Applied Physics Institute and School of Physics, Nankai University, Tianjin, 300457 China; 2 Department of Physics, Faculty of Science, University of Zagreb, Bijenička c. 32, 10000 Zagreb, Croatia; 3 Collaborative Innovation Center of Extreme Optics, Shanxi University, Taiyuan, Shanxi 030006 People’s Republic of China; 4 Center for Theoretical Physics of Complex Systems, Institute for Basic Science, Daejeon, 34126 Republic of Korea; 5 Department of Physics and Astronomy, San Francisco State University, San Francisco, CA 94132 USA

**Keywords:** Photonic crystals, Topological defects

## Abstract

Topological properties of materials are typically presented in momentum space. Here, we demonstrate a universal mapping of topological singularities from momentum to real space. By exciting Dirac-like cones in photonic honeycomb (pseudospin-1/2) and Lieb (pseudospin-1) lattices with vortex beams of topological charge *l*, optimally aligned with a given pseudospin state *s*, we directly observe topological charge conversion that follows the rule *l* → *l* + 2*s*. Although the mapping is observed in photonic lattices where pseudospin-orbit interaction takes place, we generalize the theory to show it is the nontrivial Berry phase winding that accounts for the conversion which persists even in systems where angular momentum is not conserved, unveiling its topological origin. Our results have direct impact on other branches of physics and material sciences beyond the 2D photonic platform: equivalent mapping occurs for 3D topological singularities such as Dirac-Weyl synthetic monopoles, achievable in mechanical, acoustic, or ultracold atomic systems, and even with electron beams.

## Abstract (web summary)

Topological properties of materials are typically presented in momentum space. Here, the authors show a universal mapping of topological singularities from momentum to real space, potentially applicable to a wide range of systems.

## Introduction

**Fig. 1:**
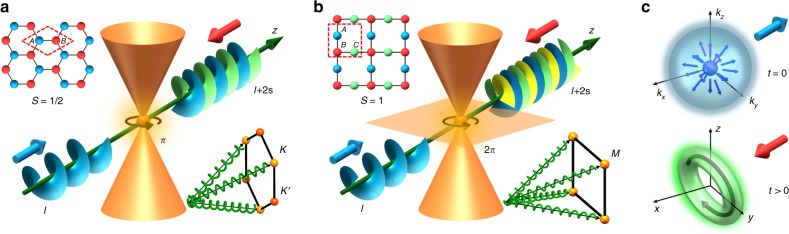
Illustration of momentum-to-real-space mapping of topological singularities. **a** A pseudospin-1/2 honeycomb lattice with two sublattices *A* and *B* is excited with three vortex beams, each with topological charge *l*. **b** A pseudospin-1 Lieb lattice with three sites (*A*, *B*, *C*) per unit cell is excited with four vortex beams. These vortex beams excite modes around conical intersections at the corners of the Brillouin zone (lower right inset). The arrows circulating around the conical intersections illustrate winding of the Berry phase (*π* in HCL and 2*π* in Lieb lattice). Topological charge conversion from *l* to *l* + 2*s* is a consequence of the mapping of topological singularity from momentum to real space. It occurs when initial excitation with *l* = ±1 is optimally aligned for a given pseudospin state (**a**) *s* = ±1/2 in HCL, and (**b**) *s* = ±1 in Lieb lattice. **c** Illustration of similar excitation of a Berry curvature monopole in 3D momentum space, leading to generation of a topological charge in real space with vorticity along the direction of excitation. Small blue and red arrows depict opposite pseudospin states.

Topological phases, as manifested in the intriguing phenomena of quantum Hall effect and topological insulators^1,2^, have attracted overwhelming transdisciplinary interest in recent years^3–7^. Topological edge states, for instance, have been realized in a variety of systems including electromagnetic waves^8–12^. Topological properties of Bloch bands are revealed in momentum space, using concepts such as the Chern number and Berry phase. We demonstrate here a universal mapping of the topology of Dirac-like cones from momentum to real space. This is achieved by properly aligned vortex excitation of pseudospin states near the Dirac-like cones in photonic honeycomb (half-integer pseudospin)^13,14^ and Lieb (integer pseudospin)^15^ lattices, leading to direct observation of topological charge conversion (as illustrated in Fig. 1a, b). We develop a unified theory to explain the observed phenomenon and present the mapping in a general topological framework involving nontrivial Berry phase winding. The topological origin of this conversion makes it both robust and universal, persisting in deformed lattices where angular momentum is not conserved, and for 3D Dirac–Weyl synthetic magnetic monopoles^16–18^ (see Fig. 1c), which can be realized in ultracold atomic gases^19^. The underlying mechanism could also be responsible for the vortex creation in electron beams traversing magnetic monopole field^20^.

The coupling of spin and orbital degrees of freedom is in many systems intertwined with the underlying topology of the space and the Berry phase^21^. For instance, in condensed matter electronic systems, studies of spin-orbit interactions led to the discovery of topological insulators, which have now emerged as an important field. The physics of electron beams illustrates many examples where spin–orbit coupling is integrated with topology^22^. There is also a plethora of related examples in optics and photonics^23^: with real space Berry phase optical elements such as q-plates and metasurfaces, circular polarization states of light (intrinsic spin) can be transformed to optical vortices carrying orbital angular momentum (OAM)^24–26^; for light propagating along a coiled ray trajectory, the dynamics is governed by the action of the monopole in Berry curvature, leading to the spin-Hall effect of light^27^. An analogous topological transport of sound waves has also been observed recently, thanks to the spin-redirection geometric phase^28^.

When discussing spin in optical systems, it is the light polarization or photon spin that is usually considered as the spin degree of freedom^23,29^. Similarly, in electronic systems it is the intrinsic electron spin^1,2^. However, for light (electrons) propagating in structured photonic media (crystalline lattices) with microscopic degrees of freedom, the concept of pseudospin independent of any intrinsic particle property emerges^13–15,30–32^. Such a concept in graphene is introduced through the mathematical analogy between the graphene sublattice degree of freedom and the electron spin in the Dirac equation. Unlike the electron spin, however, the pseudospin angular momentum is not associated with any intrinsic property of particles, but rather arises from the substructure of space (sublattices) that the particles (or wave packets) live in. For instance, the honeycomb lattice (HCL)^33^ is composed of two triangular sublattices (*A*, *B*), which features conical intersections with two touching bands at two inequivalent Dirac points (*K* and *K*’), representing a half-integer pseudospin system (*S* = 1/2, see Fig. 1a). In contradistinction, the Lieb lattice^34–36^ has three square sublattices (*A*, *B, C*), which possess a conical intersection with three touching bands at the Dirac-like *M* points, representing an integer pseudospin system (*S* = 1, see Fig. 1b).

Crucially, since the pseudospin operators satisfy a set of commutation relations directly analogous to those used to define the real spin, pseudospin should be treated on equivalent footing as other angular momenta in a given system. Consequently, a whole class of fundamental phenomena based on pseudospin-orbit interaction, twined together with topology of the underlying space, should be expected in photonic, electronic, and other relevant platforms with emergent pseudospins. We demonstrate here one such phenomenon using a photonic platform: momentum-to-real-space mapping of topological singularities.

In the paraxial approximation, light propagation in photonic lattices is governed by the Schrödinger equation^13,33^:

1 $${\rm{i}}\frac{{\partial {\mathrm{\Psi }}\left( {x,y,z} \right)}}{{\partial z}} = - \frac{1}{{2k_0}}\nabla ^2{\mathrm{\Psi }}\left( {x,y,z} \right) - \frac{{k_0{\mathrm{\Delta }}n\left( {x,y} \right)}}{{n_0}}{\mathrm{\Psi }}\left( {x,y,z} \right) \equiv H_0{\mathrm{\Psi }},$$

where Ψ is the optical field of the probe beam, *z* is the longitudinal propagation distance, *k*_0_ is the wavenumber, *n*_0_ is the background refractive index of the medium, and Δ*n* is the induced index change forming either the HCL or the Lieb lattice. In Eq. (1), *H*_0_ is the continuous Hamiltonian of the system, whose eigenvalues define the lattice band structure. For the HCL, Eq. (1) becomes a two-band simplified description of the paraxial model under the tight-binding approximation, and for excitations near the Dirac points, it turns into the linear Dirac equation typically used for describing massless Dirac particles in graphene^37^. The amplitude of the optical wave in the two sublattices is then modeled by two-component spinor functions corresponding to pseudospin. In our previous work^13^, the angular momentum associated with such lattice pseudospin has been observed. However, the topological properties arising from the interplay between pseudospin-orbit interaction and nontrivial Berry phases remain largely unexplored.

## Results

### Topological conversion in a pseudospin-1/2 honeycomb lattice

For excitations around conical intersections in both lattices, the dynamics is governed by the effective Hamiltonian

2 $$H = \kappa \left( {S_xk_x + S_yk_y} \right),$$

where *S*_*i*_ are the components of the pseudospin angular momentum operator **S**, *k*_*x*_ and *k*_*y*_ are the displacements of the transverse wavevectors with respect to the Dirac point, and *κ* depends on the properties of the lattice. The eigenstates of the pseudospin *χ*_*S*,*s*_ are given by **S**^2^*χ*_*S*,*s*_ = *S*(*S* + 1)*χ*_*S*,*s*_, and *S*_*z*_*χ*_*S*,*s*_ = *sχ*_*S*,*s*_ (here *S* and *s* denote the total and *z*-component of the pseudospin angular momentum, respectively). The eigenmodes of the Hamiltonian, *Hψ*_*n*,*k*_ = *β*_*n*,*k*_*ψ*_*n*,*k*_, are organized in 2*S* + 1 bands (labeled by *n*) touching at the conical intersection. In contrast to previous excitation schemes, we use three vortex beams (each with an initial topological charge *l* = 1 or *l* = −1) momentum-matched to the conical intersection points for the HCL (see Fig. 1a), spatially structured to excite only one pseudospin eigenstate (*s* = −1/2 or 1/2). Likewise, for the Lieb lattice, we use four vortex beams (see Fig. 1b) to excite one pseudospin eigenstate (*s* = −1, 0 or 1) for each measurement. Further experimental details about lattice creation in a 20-mm-long nonlinear crystal (SBN:61) and excitation scheme can be found in Supplementary Note 1.

**Fig. 2:**
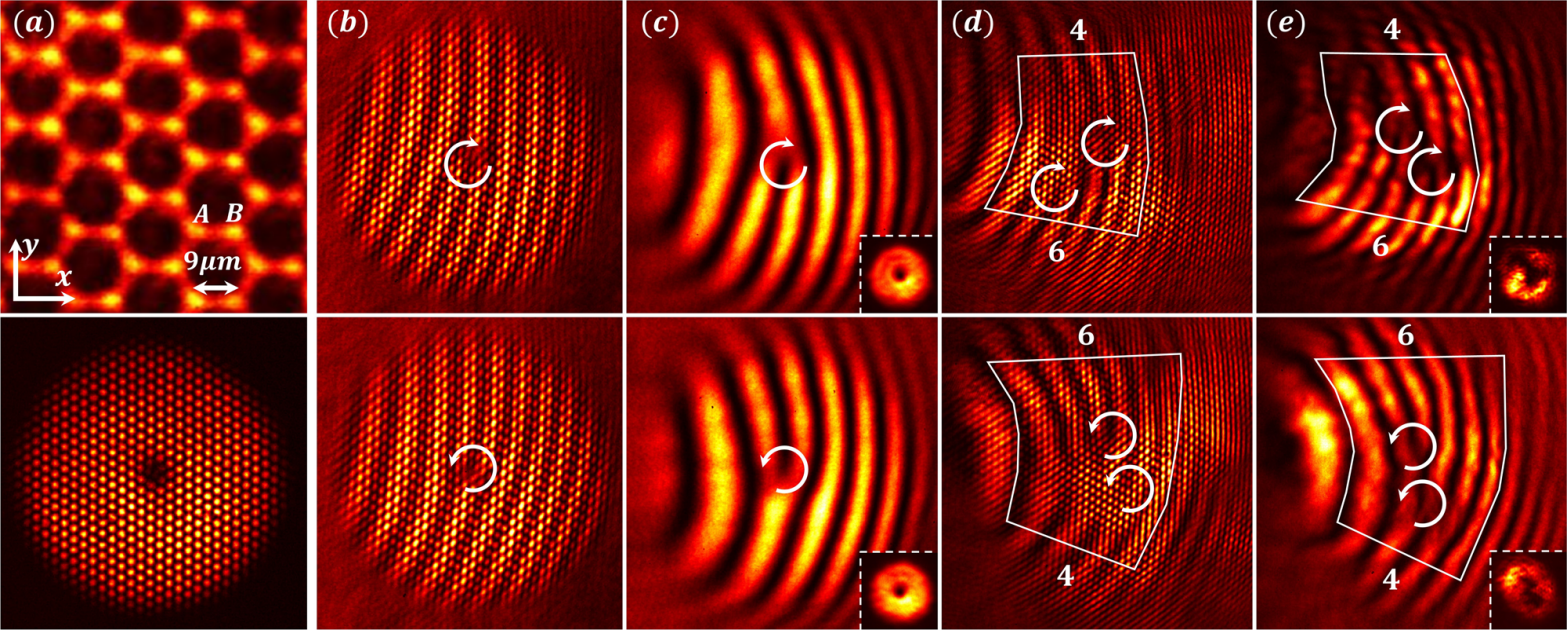
Experimental demonstration of topological conversion in a pseudospin-1/2 honeycomb lattice. **a** Top: an optically induced HCL; Bottom: input pattern of vortex-bearing triangular lattice beam used for selective excitation of the pseudospin states. **b**–**e** Top (bottom) row corresponds to initial excitation of *s* = 1/2 (*s* = −1/2) pseudospin state with vortex beams of initial topological charge *l* = 1 (*l* = −1). Interferograms of input (b, c) and output (d, e) with a tilted reference beam showing topological charge conversion from 1 to 2 (top) and from −1 to −2 (bottom). **b**, **d** Interferogram from the whole beam, and (**c**, **e**) corresponding interferogram from one of the spectral components. Difference in the numbers of counted fringes from the two sides of the marked region illustrates the net topological charges at output in (**d**, **e**). White curved arrows mark the position and helicity of the vortices. Insets in (**c**) and (**e**) show singly and doubly-charged vortex intensity patterns obtained at input and output, respectively, from one of the *K* valleys as illustrated in Fig. 1a.

Typical experimental results obtained with the HCL are summarized in Fig. 2. The HCL (Fig. 2a) is established with the multi-beam optical induction technique^38,39^. It remains invariant throughout the crystal with a nearest neighbor spacing of 9 μm. The lattice is probed by a donut-shaped triangular lattice beam, for which the OAM (*l*) and pseudospin (*s*) are optimally aligned (top panel: *l* = 1, *s* = 1/2, bottom panel *l* = −1, *s* = −1/2). To better see the phase structure of the probe beam at input (i.e., before the pseudospin-orbit interaction takes place), interferograms are obtained for the whole superimposed beam (Fig. 2b) as well as one of the three interfering beams (Fig. 2c). As illustrated in Fig. 1a, the vortex beams are momentum-matched to the three equivalent Dirac points (*K*) of the HCL. The output interferograms in Fig. 2d, e clearly display two vortices of the same helicity, which show conversion of the topological charges from *l* to *l* + 2s for both initial states (schematically illustrated in Fig. 1a). We demonstrate below that this conversion is a consequence of the mapping of topological singularity at the conical intersections from momentum to real space. Figure 2e is obtained from the Fourier transform of spectral component at one of the Dirac points back into real space for phase measurement. The bottom-right inset in Fig. 2e shows a donut-shaped intensity pattern at the output, which is somewhat deformed as compared to the input (see the inset in Fig. 2c) because it is now a higher-order vortex which tends to disintegrate into multiple singly-charged vortices during propagation in an inhomogeneous medium^40^.

It is instructive to provide a kinematical explanation of these observations (see Supplementary Note 2). In the experiment of Fig. 2, the *z*-component *J*_*z*_ of the total angular momentum **J** = **L** + **S** is conserved (where **L** = **r** × **k** is the OAM): [*J*_*z*_, *H*] = 0. The initial excitation in all experiments is comprised of a single value of *l* and *s*, and the optimally aligned initial condition implies maximal value of |*l* + *s*|. The output beam has two or more values of *l*′ and *s*′, all of which obey

3 $$l + s = {l^\prime} + {s^\prime} .$$

For example, for Fig. 2—top panel, the input beam has *l* = 1 and *s* = 1/2, while the output beam has two components: (i) *l*′ = 1 and *s*′ = 1/2, and (ii) *l*′ = 2 and *s*′ = −1/2. Since the output components are intertwined on both sublattices, we observe two vortices in Fig. 2d, e. A fully equivalent explanation holds for results in Fig. 2—bottom panel. However, as we shall discuss below through theoretical analysis, the observed charge conversion has a topological origin, holding even in systems without rotational symmetry in which angular momentum is not conserved.

**Fig. 3:**
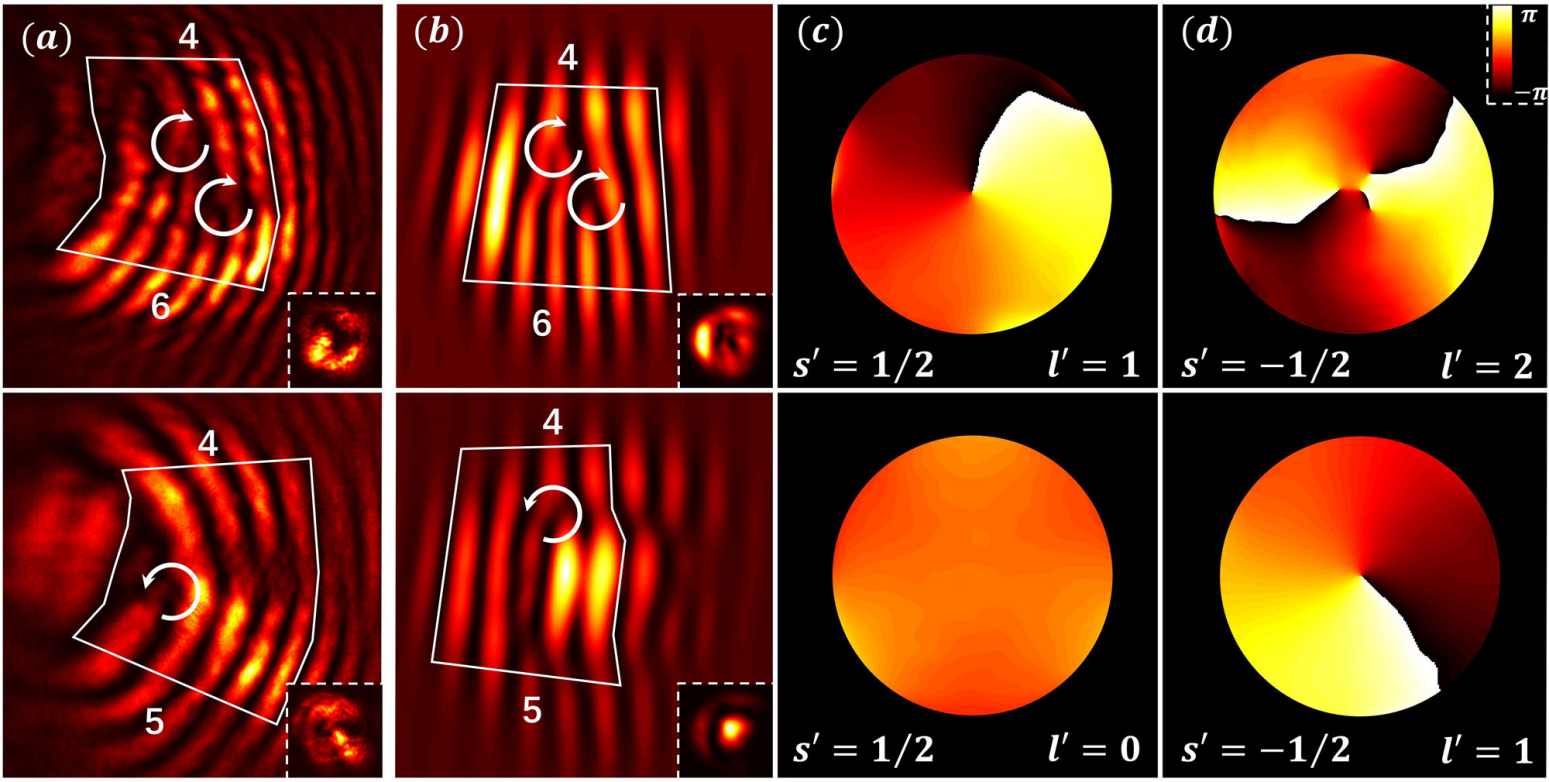
Decomposed pseudospin components in an angular-momentum-conserved HCL. Top (bottom) row: pseudospin state *s* = 1/2 (*s* = −1/2) is selectively excited with initial beam of topological charge *l* = 1. **a** Output interferogram from the experiment, and (**b**) corresponding results from the simulation with (**c**, **d**) showing the evolved phase structure separately for each pseudospin component. The topological charge increases (decreases) by 1 unit in the initially unexcited component as seen in the top (bottom) panel of **d** (**c**), while that in the initially excited component remains intact, as governed by Eq. (3). The lower insets in (**a**, **b**) are the corresponding intensity patterns; the doughnut (central bright spot) pattern corresponds to *l* ≠ 0 (*l* = 0) components.

To substantiate the above kinematical picture summarized in Eq. (3), experimental observations (Fig. 3a) are further corroborated by numerical simulations (Fig. 3b–d) based on the paraxial wave equation (Eq. (1)). For all simulations, the parameters are chosen close to those from experiment with the index contrast *δn* = 2 × 10^−4^ .We excite the pseudospin states *s* = 1/2 (top) and *s* = −1/2 (bottom), with the input beam of topological charge *l* = 1 covering sublattices *A* and *B*, respectively. In numerical simulations, the output field is decomposed into each pseudospin component. From the phase structure of each component, the difference is clear: if the *s* = 1/2 component is initially excited, the unexcited *s*′ = −1/2 component is converted into an *l*′ = 2 vortex (Fig. 3d, top). In contrast, if the *s* = −1/2 is initially excited, the vorticity in the unexcited *s*′ = 1/2 component disappears, *l*′ = 0 (Fig. 3c, bottom). The vorticity of the initially excited component always remains unchanged, in accordance with Eq. (3). Note that the output intensity patterns in the lower insets of Fig. 3 have a subtle difference between the two cases of excitation: the donut shape is preserved in the top panels (when both components maintain a vortex), but deforms to have a bright central spot in bottom panels (when vortex annihilation occurs in one of the components).

### Topological conversion in a pseudospin-1 Lieb lattice

**Fig. 4:**
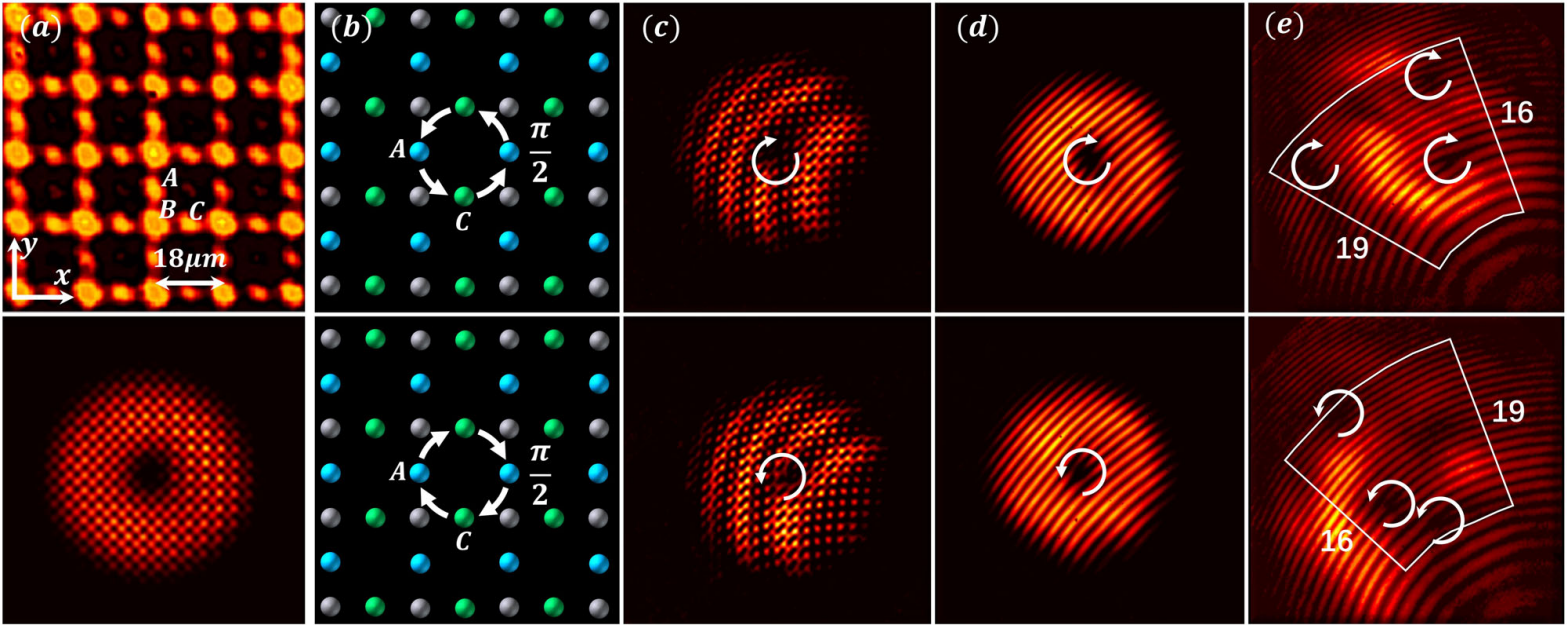
Experimental demonstration of topological conversion in a pseudospin-1 Lieb lattice. **a** Top: an optically induced Lieb lattice; Bottom: input pattern of vortex-bearing square lattice beam used to excite the pseudospin states of the Lieb lattice. **b** Top (bottom) row illustrates selective excitation of *A* and *C* sublattices with an appropriate phase relation optimized for pseudospin states *s* = 1 (*s* = −1) by vortex beams of initial topological charge *l* = 1 (*l* = −1). **c**–**e** Interferograms of input (**c**, **d**) and output (**e**) showing topological charge conversion from 1 to 3 (top) and from −1 to −3 (bottom).

Next, we discuss experimental results obtained with a photonic Lieb lattice (summarized in Fig. 4). The lattice is established again by optical induction in the 20-mm-long crystal^36^, with a nearest neighbor spacing of 9 μm. A donut-shaped square lattice beam (Fig. 4a, bottom) created by interfering four singly-charged vortex beams is employed as a probe, which excites only one pseudospin component (top panel: *l* = 1, *s* = 1; bottom panel: *l* = −1, *s* = −1). In the Lieb lattice, the pseudospin *S*_*z*_ is not diagonal in sublattice basis^15^. Therefore, to excite a given pseudospin state, the probing square lattice is matched either to the *B* sublattice (for the *s* = 0 pseudospin state) or the *A* and *C* sublattices with appropriate phase relation (for the *s* = 1 and *s* = −1 pseudospin states) as illustrated in Fig. 4b. The output interferograms in Fig. 4e clearly display three vortices of the same helicity as the input (i.e., a net topological charge of 3 or −3), exhibiting a conversion of the topological charges from *l* to *l* + 2s for the optimally aligned excitations (schematically illustrated in Fig. 1b).

**Fig. 5:**
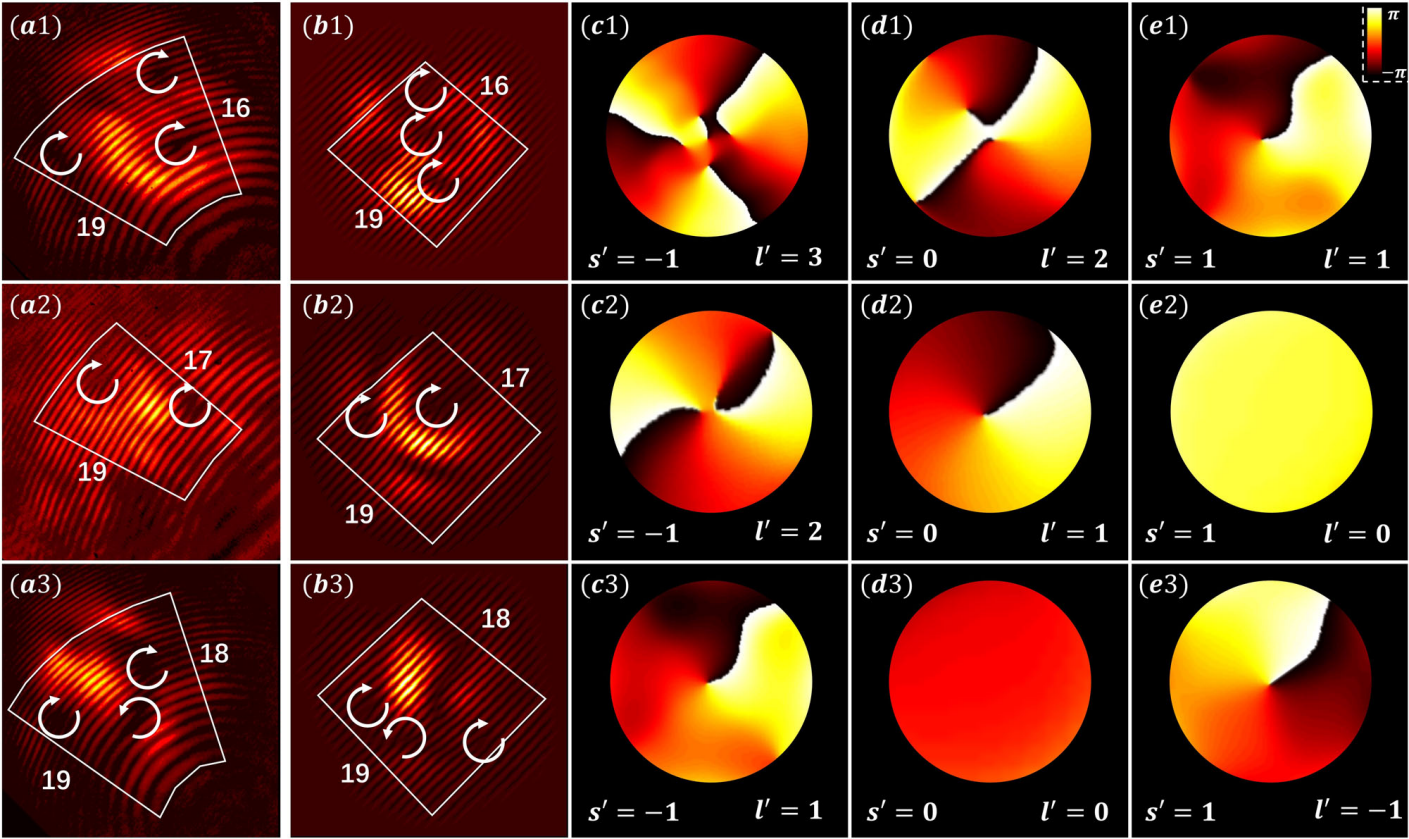
Decomposed pseudospin components in an angular-momentum-conserved Lieb lattice. Experiment and simluation results for initial excitations of the pseudospin states *s* = 0 in (a1–e1), *s* = 1 in (a2–e2) and *s* = −1 in (a3–e3) with four input beams of topological charge *l* = 1. Output interfrograms from (**a**) experiment and (**b**) simulation show different topological charge conversions under different excitation conditions. (**c**–**e**) show output phase structure of the probe beam numerically decomposed for each pseudospin component *s*′, where corresponding output vorticity *l*′ in each component has been identified. In all cases, each pseudospin component obeys Eq. (3).

In Fig. 5, we show experimental (Fig. 5a) and numerical (Fig. 5b) results obtained by initial excitation of the pseudospin states (from top to bottom rows) *s* = 1, *s* = 0, and *s* = −1 with a proper input beam of topological charge *l* = 1, and examine how the phase evolves for the three decomposed pseudospin components (Fig. 5c–e). The first case (*s* = 1) corresponds to optimally aligned excitation in Fig. 4, where the topological charge emerging in the *s*′ = −1 pseudospin component is *l*′ = 3. For the latter two cases (*s* = 0, and *s* = −1), which are not optimally aligned, the initial vortex is also transformed into multiple vortices but with a net topological charge of 2 (middle row) or 1 (bottom row), while all components satisfy again the kinematics of pseudospin-orbit interaction (Eq. (3)). Similar studies with the input beam of topological charge *l* = −1 led to the same conversion rule. The pseudospin components of the Lieb lattice are not diagonal in the sublattice basis, and therefore do not have a trivial correspondence to a particular sublattice^15^ as for the case of pseudospin-1/2 HCL^13^. In fact, the physics of pseudospin-orbit interaction in Lieb lattices is in one aspect richer than that of polarization-based spin–orbit interaction: here we have excited also the *s* = 0 pseudospin state, inadmissible for helicity of photons due to its zero mass.

### Fundamental mechanism of the topological charge conversion

Up to this point, we have discussed topological charge conversion in honeycomb and Lieb lattices and explained the results kinematically with angular momentum conservation. One can also explain the observed phenomenon dynamically, by expanding the initial excitation in eigenmodes of the Hamiltonian, as elaborated in the Methods section. However, there is a fundamental topological mechanism beyond the charge conversion observed in experiment. When eigenmodes of the Hamiltonian (2) are expanded in pseudospin eigenstates, there are vortices (i.e., topological charges) in the *k*-space attached to the components, and the topological charge of neighboring pseudospin components differs by one. For example, for the HCL the eigenmodes are $$\psi _{n,\;{\mathbf{k}}} = \frac{1}{{\sqrt 2 }}\left( {\begin{array}{*{20}{c}} n \\ {{\rm{e}}^{{\rm{i}}\varphi _k}} \end{array}} \right) = \frac{1}{{\sqrt 2 }}\left( {n\chi _{\frac{1}{2},\frac{1}{2}} + {\rm{e}}^{{\rm{i}}\varphi _k}\chi _{\frac{1}{2}, - \frac{1}{2}}} \right)$$ (see Methods and Supplementary Notes). When a single pseudospin component is excited, the *k*-space vortex in the other component is mapped from momentum to real space, giving rise to topological charge conversion. Difference in the *k*-space topological charges of pseudospin components is related to the Berry phase winding around the Dirac point (see Methods). If we denote the winding of the Berry phase around the Dirac point with *wπ*, then for the HCL, *w* = 1; and for the Lieb lattice, *w* = 2. Topological quantity *w* is also the maximal difference between the *k*-space topological charges of pseudospin components. For the studied honeycomb and the Lieb lattices, the rule *l* → *l* + 2*s*, which holds only for optimally aligned excitations, can be expressed as *l* → *l* + *w* for *l* > 0 (or *l* → *l*−*w *for *l* < 0). It turns out that this latter expression of the conversion rule, which contains the topological quantity *w*, is more general than the one containing pseudospin *s*, as can be viewed on the following examples.

**Fig. 6:**
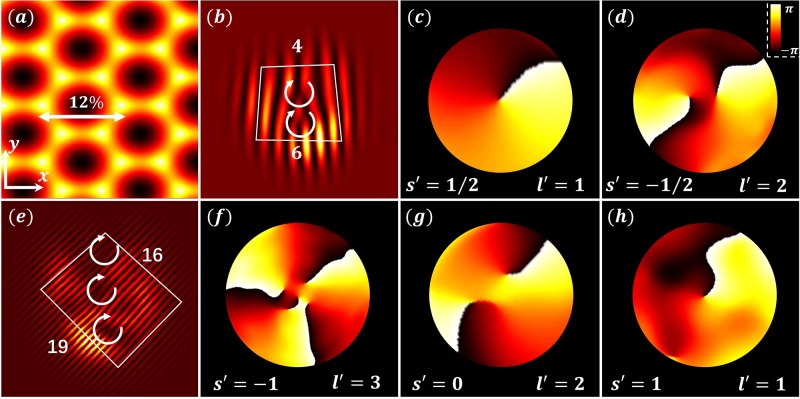
Evolution of pseudospin states in stretched lattices lacking rotational symmetry around conical intersections. Top (bottom) row corresponds to a stretched HCL (Lieb lattice), with an initial excitation *l* = 1, *s* = 1/2 (*l* = 1, *s* = 1). **a** A 12% horizontally stretched HCL. **b**, **e** Interferograms of the output beam, which clearly indicate the topological conversion from *l* to *l* + 2s (or *l* ± *w*), corresponding to results from unstretched lattices of the top rows in Figs. 3b and 5b. This is underpinned by the phase structure of the pseudospin components at the output illustrated in (**c**, **d**) and (**f**–**h**) for the two lattices.

Consider a conical intersection described by the Hamiltonian *H*_*s*_ = *κ*_*x*_*S*_*x*_*k*_*x*_ + *κ*_*y*_*S*_*y*_*k*_*y*_, where the angular momentum is not conserved for *κ*_*x*_ ≠ *κ*_*y*_ due to lack of rotational symmetry; an inspection of the eigenstates of *H*_*s*_ for pseudospin *S* = 1/2 and *S* = 1 shows that the *k*-space vortices become elliptical but preserve their topological charge. For the stretched HCL, the winding of the Berry phase around the Dirac point is protected, until the stretching is sufficiently large so that the inequivalent Dirac points merge and a gap opens^41–43^. After this transition the Berry phase vanishes and the topological charge conversion no longer occurs. In Fig. 6 we show numerical simulations for the optimally aligned initial condition in the stretched HCL and Lieb lattice. The nearest neighbor spacing for the HCL and the Lieb lattice is 9 µm, stretched by 12% and 15%, respectively. As seen from these results, the conversion of the topological charges from *l* to *l* ± *w* holds even when the angular momentum *J*_*z*_ is not conserved, which indicates that the mapping of the topological charges from *k*-space to *x*-space is a fundamental process with topological origin. Other examples include band structures with parabolic band-touching points, or conical intersections described by the Hamiltonian *H*_0_ = *kσ*_*z*_, where the formula *l* → *l* ± *w* still holds, further underpinning the topological interpretation of our results (see Methods and Supplementary Notes).

Next, we expand our results beyond the 2D platform. Our experiments are performed in 2D lattices; however, analogous considerations can be made for 3D Hamiltonians. For example, we consider the Weyl Hamiltonian $H_{{\mathrm{Weyl}}} = {\mathbf{\sigma }} \cdot {\mathbf{k}}$, which has attracted considerable interest in recent years; it has been experimentally realized in the Brillouin zone of specially designed optical and condensed matter structures^16,17^. This Hamiltonian gives rise to a synthetic magnetic monopole in *k*-space^18^, with topological charge 1. Suppose that we initially excite the modes around a Weyl point with a symmetric Gaussian-like distribution, and that we excite a pseudospin eigenstate $\chi _{\frac{1}{2},\frac{1}{2}}$. The initial state is $\psi _{0,\frac{1}{2}}\left( {r,\theta _r,\varphi _r,t = 0} \right) = \psi _0(r)\chi _{\frac{1}{2},\frac{1}{2}}$, and its evolution in time is governed by ${\rm{i}}\frac{{\partial \psi }}{{\partial t}} = H_{{\mathrm{Weyl}}}\psi$. By expanding the initial state in eigenmodes of the Weyl Hamiltonian, it is straightforward to see that the wavefunction evolves as (see Supplementary Notes for details)

4 $$\psi _{0,\frac{1}{2}}\left( {r,\theta _r,\varphi _r,t} \right) = \chi _{\frac{1}{2},\frac{1}{2}}g_{\frac{1}{2},\frac{1}{2}}\left( {r,\theta _r,t} \right) + {\rm{e}}^{{\rm{i}}\varphi _r}\chi _{\frac{1}{2}, - \frac{1}{2}}g_{\frac{1}{2}, - \frac{1}{2}}\left( {r,\theta _r,t} \right).$$

Clearly, even though the initial state was a Gaussian-like excitation with *l* = 0 and *s* = 1/2, in the unexcited pseudospin component *s*′ = −1/2, a vortex with topological charge *l*′ = 1 emerges, as illustrated in Fig. 1c. Since this is a 3D rotationally invariant Hamiltonian, [**J**, *H*_Weyl_] = 0, if we excite any pseudospin state $\alpha \chi _{\frac{1}{2},\frac{1}{2}} + \beta \chi _{\frac{1}{2}, - \frac{1}{2}}$ with a Gaussian-like distribution, we will obtain in the output a vortex field with a topological charge identical to the charge of the Weyl monopole, with vorticity pointing in the direction of the initial pseudospin. Thus, with properly designed initial excitation, mapping of topological properties of the Weyl monopole to topological charges in real space can be readily achieved. In fact, this type of dynamics in 3D Weyl systems, achievable in ultracold atomic gases^19^, seems to be related to a recent experiment where an electron beam scattered from a magnetic monopole experienced a conversion into an electron vortex^20^.

We have thus demonstrated the universal mapping of topological singularities in *k*-space to measurable topological entities in real space. Our experiments were carried out in photonic honeycomb and Lieb lattices, where the mapping can be explained with pseudospin-orbit interaction, angular momentum conservation and nontrivial winding of the Berry phase. However, we have demonstrated in theory that the underlying mechanism for the mapping lies in fundamentally topological origin. Besides the typical honeycomb and Lieb conical intersections, we have shown that it occurs also in stretched lattices where angular momentum is not conserved, and for parabolic band touching and other nonconical intersections. Moreover, we have predicted that the same mechanism exists in 3D Weyl lattices where synthetic magnetic monopoles come to play the role. Our finding brings about many interesting questions as well as opportunities. For instance, is it possible to create vortices of Bose–Einstein condensates^44^ by topological conversion from synthetic magnetic monopoles in ultracold atomic gases^19^? How could the mechanism explored here be adapted for topological conversion with photons in a photonic Dirac monopole field^45^? It is also natural to ask: is the spin angular momentum gifted by light polarization indispensable in spin-to-orbital angular momentum conversion, as commonly thought, or the pseudospin and topological conversion is essential even in those conventional settings based on optical phase elements^24,26^? What other mechanisms can we conceive and explore where topological properties of the bands can be directly mapped from momentum to real space in experiments? Can other topological entities such as vortex knots and nodal chains^46–48^ be directly mapped from momentum space to real space or vice versa, or onto a synthetic space^49,50^?

## Methods

### Theoretical framework for topological conversion

We develop here a systematic theoretical framework to fully analyze the observed phenomena, which unravels the connections between pseudospin, OAM and the underlying topology of the lattice in *k*-space. The initial vortex beam which probes the conical intersection of Hamiltonian in Eq. (2) is described with the complex amplitude of the electric field $\psi _{l,s}(r,\varphi _r,z = 0) = \psi _0r^l{\rm{e}}^{{\rm{i}}l\varphi _r}\exp \left( { - r^2\!/\!a_0^2} \right)\chi _{S,s}$, where *χ*_*S*,*s*_ accounts for the fact that we initially excite only a single pseudospin component. The probe beam is broad in real space (*a*_0_ ≫ lattice constant), and narrow in momentum space. We expand the initial excitation in eigenmodes of the Hamiltonian (2) and account for the dynamics via $\psi _{l,s}\left( {r,\varphi _r,z} \right) = \mathop {\sum}\nolimits_{n,{\mathbf{k}}} {c_{n,{\mathbf{k}}}} \psi _{n,{\mathbf{k}}}{\mathrm{exp}}\left( -{{\mathrm{i}}\beta _{n,{\mathbf{k}}}z} \right)$; the coefficients *c*_*n*,*k*_ are found by calculating projection 〈*ψ*_*n*,*k*_|*ψ*_*l*,*s*_(*z* = 0)〉 (see Supplementary Notes). For the optimally aligned initial state in HCL (e.g., exciting the *s* = 1/2 state with *l* = 1), it is straightforward to find the evolving complex amplitude of the electric field:

5 $$\psi _{l = 1,s = \frac{1}{2}}\left( {r,\varphi _r,z} \right) = {\rm{e}}^{{\rm{i}}l\varphi _r}\chi _{\frac{1}{2},\frac{1}{2}}g_{\frac{1}{2},\frac{1}{2}}\left( {r,z} \right) + {\rm{e}}^{{\rm{i}}\left( {l + 1} \right)\varphi _r}\chi _{\frac{1}{2}, - \frac{1}{2}}g_{\frac{1}{2}, - \frac{1}{2}}\left( {r,z} \right).$$

The radial and *z*-dependence of the electric field is for clarity denoted with $g_{\frac{1}{2}, \pm \frac{1}{2}}\left( {r,z} \right)$, and it produces the conical diffraction pattern^13,33^. This is in full agreement with observations in Figs. 2 and 3 (top rows), which show that the initially unexcited *s*′ = −1/2 component has vorticity *l*′ = *l* + 1, whereas the excited *s*′ = 1/2 component has vorticity *l*′ = *l*.

In a fully equivalent manner, we can describe the dynamics in the Lieb lattices. For the optimally aligned initial excitation (e.g., exciting the *s* = 1 state with *l* = 1), the electric field evolves according to the following (see Supplementary Notes):

6 $$\psi _{l = 1,s = 1}\left( {r,\varphi _r,z} \right) = {\rm{e}}^{{\rm{i}}l\varphi _r}\chi _{1,1}g_{1,1}\left( {r,z} \right) \! + {\rm{e}}^{{\rm{i}}\left( {l + 1} \right)\varphi _r}\chi _{1,0}g_{1,0}\left( {r,z} \right) + {\rm{e}}^{{\rm{i}}(l + 2)\varphi _r}\chi _{1, - 1}g_{1, - 1}\left( {r,z} \right).$$

We see that the topological charge emerged in the pseudospin components *s* = 0 and −1 is *l* + 1 and *l* + 2, respectively, in accordance with experimental results and numerical simulations presented in Figs. 4 and 5 (top rows), and the kinematical arguments. Dynamical considerations provide, in addition, the details of the *r*- and *z*-dependence of the electric field, which are contained in the g-functions. For all other initial conditions, similar calculations yield results also in accordance with observations.

Although the above dynamical explanation seems enough to address the observed phenomena, there is a fundamental connection to the underlying topology of *k*-space and the observation of vortices in *x*-space. If a beam propagates sufficiently long in a photonic lattice, as in our experiments, the output intensity of the beam in *x*-space will reflect the initial distribution of the power in the lattice *k*-space (analogous to far-field dynamics in free space). However, the HCL possesses topological singularity at the Dirac point: the Berry phase acquired as one traverses a loop around the Dirac point is *π*; the Dirac point can be considered as a flux tube (topological singularity) of the Berry curvature^51^. Our experiments essentially reveal how the excitations of modes around the singularity are mapped into the far field dynamics.

To see that clearly, we revisit the calculation of the Berry phase in the HCL. An HCL eigenstate close to the conical intersection can be written as $$\psi _{n,{\mathbf{k}}} = \frac{1}{{\sqrt 2 }}\left( {\begin{array}{*{20}{c}} n \\ {{\rm{e}}^{{\rm{i}}\varphi _k}} \end{array}} \right)$$, with *n* = ±1. As we adiabatically circle around the Dirac point, the acquired Berry phase is $- {\rm{i}}{\oint} {\left\langle {\psi _{n,{\mathbf{k}}}\left| {\frac{\partial }{{\partial \varphi _k}}} \right|\psi _{n,{\mathbf{k}}}} \right\rangle } {\rm{d}}\varphi _k = \pi$. The Berry phase arises from the specific phase relation in *k*-space between the pseudospin components of the eigenstate. There is a vortex (i.e., a topological charge) in *k*-space in one of the pseudospin components; more precisely, the difference in *k*-space topological charges of the two components is one. From the derivation of Eq. (5), we see that during propagation this vortex is mapped from the *k*-space to the *x*-space (see Supplementary Notes). Thus, what we observed in our experiments is the topological singularity of the HCL mapped from momentum to real space. The mapping is revealed due to the properly designed initial excitation of a single pseudospin state.

This finding holds for a general Hamiltonian (2), i. e., for any pseudospin *S*. Every eigenmode can be expanded in pseudospin eigenstates as $\psi _{n,{\mathbf{k}}} = \mathop {\sum}\nolimits_{s = - S}^S {\left\langle {\chi _{S,s}{\mathrm{|}}\psi _{n,{\mathbf{k}}}} \right\rangle } \chi _{S,s}$. The coefficients 〈*χ*_*S*,*s*_|*ψ*_*n*,**k**_〉 are found by rewriting the Hamiltonian as $H = \frac{\kappa }{2}k\left( {S_ + {\rm{e}}^{ - {\rm{i}}\varphi _k} + S_ - {\rm{e}}^{{\rm{i}}\varphi _k}} \right)$, where *S*_±_ = *S*_*x*_ ± i*S*_*y*_:

7 $$\beta _{n,{\mathbf{k}}}\left\langle {\chi _{S,s}{\mathrm{|}}\psi _{n,{\mathbf{k}}}} \right\rangle =	 \,\, \left\langle {\chi _{S,s}{\mathrm{|}}H{\mathrm{|}}\psi _{n,{\mathbf{k}}}} \right\rangle \\ =	 \,\, \frac{\kappa }{2}k\left( \sqrt {\left( {S - s} \right)\left( {S + s + 1} \right)} \left\langle {\chi _{S,s + 1}{\mathrm{|}}\psi _{n,{\mathbf{k}}}} \right\rangle {\rm{e}}^{ - {\rm{i}}\varphi _k} \right. \\ \, 	+ \left. \sqrt {\left( {S + s} \right)\left( {S - s + 1} \right)} \left\langle {\chi _{S,s - 1}{\mathrm{|}}\psi _{n,{\mathbf{k}}}} \right\rangle {\rm{e}}^{{\rm{i}}\varphi _k} \right).$$

There is a clear phase relationship between different pseudospin components of the eigenstates. The difference in *k*-space topological charges (vortices) of neighboring pseudospin components is one. When a single pseudospin component is excited, *k*-space topological charges of the unexcited components are mapped to real space, which is the fundamental mechanism behind topological charge conversions observed in our experiments.

To further corroborate the topological interpretation of the conversion, let us consider the following two examples. The first is the Hamiltonian

8 $$H_m = \left( {\begin{array}{*{20}{c}} 0 & {\left( {k_x - {\rm{i}}k_y} \right)^m} \\ {\left( {k_x + {\rm{i}}k_y} \right)^m} & 0 \end{array}} \right),$$

which for *m* = 1 corresponds to the HCL we studied above. The band structure for *m* = 2 corresponds to bilayer graphene and has a parabolic band-touching point, and for *m* > 2 it corresponds to other variants of band touching. It is straightforward to verify that if the nonconical Dirac-like points are excited with a fully equivalent optimal excitation as for the HCL, the topological charge conversion *l* → *l* ± *w* still holds. However, the expression *l* → *l* + 2 *s* relying on the angular momentum is no longer applicable. Note that for the Lieb and bilayer graphene lattices, the topological conversion arises from the Berry phase of 2*π*, which may seem trivial at glance^52^. In a similar fashion, one may explore another example of the Hamiltonian *H*_0_ = *kσ*_*z*_, which has a conical intersection point like the HCL case but here with *w* = 0. It can be shown again that *l* → *l* ± *w* is valid, consolidating our finding that a more general interpretation of our experimental observations should be formulated by the winding of the Berry phase as the topological quantity, rather than just the pseudospin.

## Supplementary information

Supplementary Information

## Data Availability

The data that support the findings of this study are available from the corresponding author upon reasonable request.
