# Supplementary material for: Universal momentum-to-real-space mapping of topological singularities
Source: Nat Commun. 2020 Mar 27;11:1586. doi: 10.1038/s41467-020-15374-x (PMC7101314; doi:10.1038/s41467-020-15374-x)
Supplement: Supplementary file 1 — Supplementary Information [file 41467_2020_15374_MOESM1_ESM.pdf]

Supplementary Information:

Universal momentum-to-real-space mapping of topological singularities

Xiuying Liu[1+], Shiqi Xia[1+], Ema Jajtić[2+], Daohong Song[1,3*], Denghui Li[1], Liqin Tang[1,3], Daniel Leykam[4], Jingjun Xu[1,3], Hrvoje Buljan[1,2*], and Zhigang Chen[1,3,5*]

[1]The MOE Key Laboratory of Weak-Light Nonlinear Photonics, TEDA Applied Physics Institute and School of Physics, Nankai University, Tianjin 300457, China
[2]Department of Physics, Faculty of Science, University of Zagreb, Bijenička c. 32, 10000 Zagreb, Croatia
[3]Collaborative Innovation Center of Extreme Optics, Shanxi University, Taiyuan, Shanxi 030006, People's Republic of China
[4]Center for Theoretical Physics of Complex Systems, Institute for Basic Science, Daejeon 34126, Republic of Korea
[5]Department of Physics and Astronomy, San Francisco State University, San Francisco, California 94132, USA
**Corresponding authors: songdaohong@nankai.edu.cn, hbuljan@phy.hr, zgchen@nankai.edu.cn*
[+]These authors made equal contribution.

Supplementary Note 1:

<u>Experiment setup and scheme for pseudospin excitation:</u> The experimental setup for generation of optically induced photonic lattices is illustrated in Supplementary Figure 1. Both the HCL and the Lieb lattices are created by sending a modulated lattice intensity pattern propagating invariantly throughout a 20mm-long negatively biased photorefractive SBN crystal, which turns into a refractive index pattern (i.e., the lattice) under the action of nonlinearity [1,2]. For the HCL, an amplitude mask is used, which turns the input pattern into a triangular lattice beam, as illustrated in the inset of path 1. When a voltage is applied against the crystalline *c*-axis, the lattice beam experiences a self-defocusing nonlinearity which transforms the triangular intensity pattern into the HCL index potential [1]. By use of an SLM loaded with a desired phase pattern

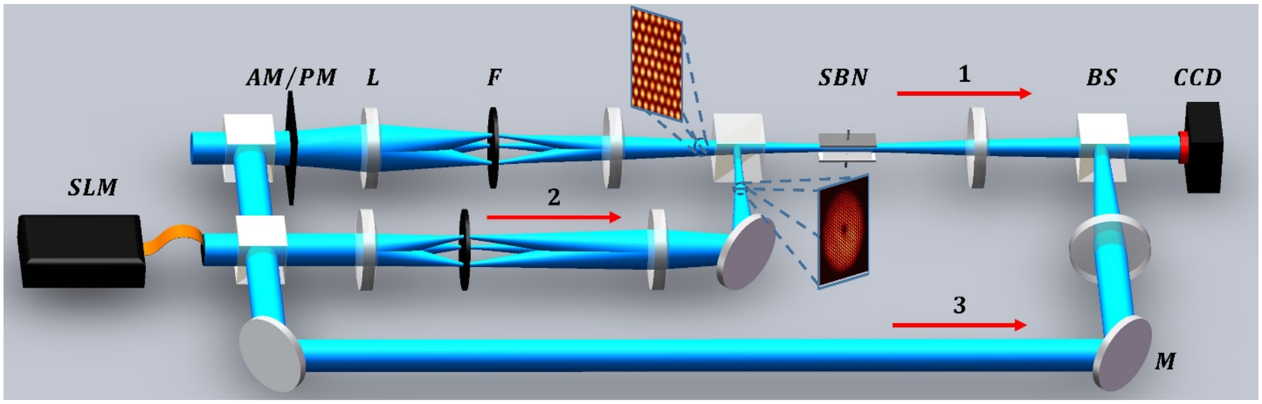

Supplementary Figure 1. Experimental setup for optical induction of photonic HCL and Lieb lattices in a nonlinear crystal. SLM: spatial light modulator; AM/PM: amplitude /phase mask; L: lens; F: Fourier filter; BS: beam splitter; SBN: strontium barium niobite crystal; M: mirror. Path 1 is for the ordinarily-polarized lattice-induction beam after amplitude/phase modulation, path 2 is for the extraordinarily-polarized probe beam (interference of multi-vortex beams created from SLM), and path 3 is for the reference beam (also extraordinarily-polarized) used to measure the output phase. The inset in path 1 illustrates a triangular (square) lattice beam pattern used for "writing" the honeycomb (Lieb) photonic lattice, and the inset in path 2 shows the intensity pattern of the vortex probe beam.

for three vortex beams with same topological charge l, the probe beam forms a donut-shaped triangular lattice pattern also with a net topological charge l (see Fig. 2a) before going to the crystal to probe the lattice. In momentum space, the directions of three vortex beams are matched to the three Dirac K points of the first Brillouin zone, while in real space, the triangular intensity pattern is matched to A or B sublattice to selectively excite the two pseudospin states, as illustrated in Fig. 1(a).

For the Lieb lattice, a slightly different technique is used for optical induction. A phase mask (in this case, an SLM) is used to generate two square lattice beams with different periods ($9\mu m$ and $18\mu m$), and then the two induced index lattices are superimposed to form the Lieb lattice under the self-defocusing nonlinearity [2]. For probing, four vortex beams with same topological charge l are employed, which form a donut-shaped square lattice pattern also with a net topological charge l (see Fig. 4a). However, due to the non-diagonal nature of pseudospin-1 Hamiltonian, the three Lieb sublattices do not have a trivial correspondence to the three pseudospin states [illustrated in Supplementary Figure2(a)] as for the case of the HCL. As such, we elaborate here the excitation scheme for the Lieb lattice. After the Lieb lattice is written in the crystal, the four vortex beams are matched in momentum space to the four M points at the corner of first Brillouin zone but with different phase winding [Supplementary Figure 2(b)]. This phase winding shows also different phase relation between the excited lattice sites in real space

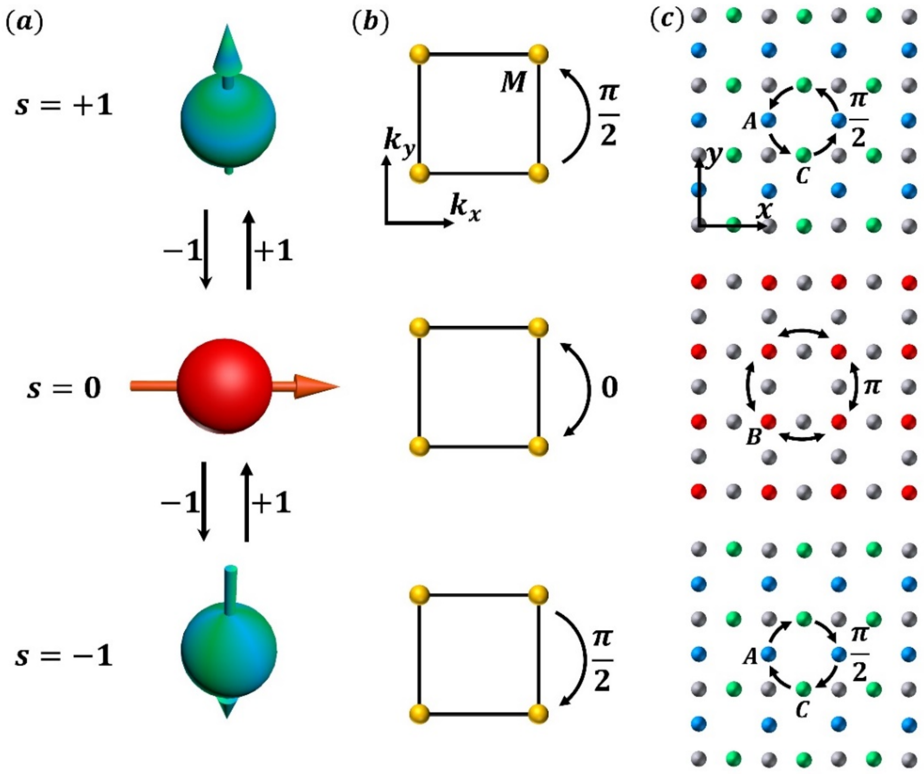

Supplementary Figure 2. Illustration of excitation scheme for different pseudospin states in Lieb lattice. Top row: excitation condition for $s = +1$. (a) Illustration of the pseudospin $s = +1$ component aligned along S_z. (b) Momentum space illustration of the four vortex beams as a lattice probe matched to the four M points with an anti-clockwise $\pi/2$ phase winding. (c) Real space illustration of selective excitation of A and C sublattices with the phase winding marked by arrows. Sites with zero amplitudes are denoted by gray dots, and those with nonzero amplitudes by colored dots. Middle and bottom rows are excitation conditions for $s = 0$ and $s = -1$, respectively.

[Supplementary Figure2(c)]. To excite the $s = 0$ pseudospin state, the square lattice pattern of the probe beam is matched only to the B sublattice, with a π phase difference between the nearest excited sites. However, to excite the $s = 1$ and $s = -1$ pseudospin states, the square lattice pattern is matched to excite simultaneously A and C sublattices but with an opposite phase winding in a π/2 phase step. This phase requirement used in our experiment agrees perfectly with the eigenstates of the pseudospin matrix S_z, as shown in the following theory section for the Lieb lattice.

Supplementary Note 2:

Details of theoretical derivation: The photonic honeycomb and Lieb lattices that we consider here have conical intersection points in k-space (e.g., see [1-5] and references therein). For excitations in the vicinity of these points, the dynamics is governed by the Hamiltonian

$$H = \kappa\left(S_x k_x + S_y k_y\right) \tag{1}$$

where S_i are the components of the pseudospin angular momentum operator $\mathbf{S}$, which obey angular momentum commutation relations:

$$[S_a, S_b] = i\varepsilon_{abc} S_c,$$

where ε_{abc} is the Levi-Civita symbol; the constant κ depends on the specific properties of the lattice. The eigenstates of the pseudospin are given by $\mathbf{S}^2\chi_{S,s} = S(S+1)\chi_{S,s}$, and $S_z\chi_{S,s} = s\chi_{S,s}$. The Hamiltonian (Supplementary Equation 1) has $2S+1$ bands, i.e., the honeycomb lattice has 2 bands, while the Lieb lattice has 3 bands. As in the main text, we denote z-components of the angular momenta with lower case letters.

Conservation of the angular momentum: The kinematical explanation of our observations involves conservation of the z-component J_z of the total angular momentum $\mathbf{J} = \mathbf{L} + \mathbf{S}$, where $\mathbf{L} = \mathbf{r} \times \mathbf{k}$ is the orbital angular momentum (OAM). The following commutation relations hold:

$$[L_a, L_b] = i\varepsilon_{abc} L_c,\ \ [S_a, L_b] = [S_a, k_b] = 0,\ \ [k_a, L_b] = i\varepsilon_{abc} k_c.$$

It is straightforward to demonstrate $[J_z, H] = 0$:

$$\frac{1}{\kappa}[H, J_z] = \left[\left(S_x k_x + S_y k_y\right), L_z + S_z\right] = S_x[k_x, L_z] + [S_x, L_z]k_x + S_y\left[k_y, L_z\right] + \left[S_y, L_z\right]k_y + S_x[k_x, S_z] + [S_x, S_z]k_x + S_y\left[k_y, S_z\right] + \left[S_y, S_z\right]k_y = -iS_x k_y + 0 + iS_y k_x + 0 + 0 - iS_y k_x + 0 + iS_x k_y = 0,$$

which indicates that the z-component of the total angular momentum is conserved.

The initial excitations in our experiments are comprised of a single value of l and s. We use $l = 1$ or $l = -1$ for the input. The admissible values of pseudospin (z-component) are $s =$

$-S, -S+1, \cdots, S-1, S$. Thus, we study 4 possible initial conditions for the HCL, and 6 possible initial conditions for the Lieb lattice. Maximally aligned initial condition implies maximal value of $|j| = |l+s|$, and there are 2 such conditions for each lattice. The output beam has two or more values of l' and s', all of which obey $l+s = l'+s'$ due to $[J_z, H] = 0$, as expected in an angular momentum conserved system.

Honeycomb lattice – dynamics via expansion over the eigenstates: Now we derive Eq. (5) in the main text, i.e., we account for the dynamics via expansion over the eigenstates. First, we consider graphene-like honeycomb lattices where $S = 1/2$, $\mathbf{S} = \boldsymbol{\sigma}/2$, and $\boldsymbol{\sigma}$ are the Pauli matrices. The Hamiltonian is

$$H = \frac{\kappa}{2}\left(\sigma_x k_x + \sigma_y k_y\right), \tag{2}$$

and the Pauli matrices are:

$$\sigma_x = \begin{pmatrix} 0 & 1 \\ 1 & 0 \end{pmatrix}, \sigma_y = \begin{pmatrix} 0 & -i \\ i & 0 \end{pmatrix}, \text{and } \sigma_z = \begin{pmatrix} 1 & 0 \\ 0 & -1 \end{pmatrix}.$$

The eigenvalues (propagation constants) of the Hamiltonian (Supplementary Equation 2) are,

$$\beta_{n,\mathbf{k}} = n\frac{\kappa}{2}\sqrt{{k_x}^2 + {k_y}^2} = n\frac{\kappa}{2}k,$$

and the eigenmodes are:

$$\psi_{n,\mathbf{k}} = \frac{1}{\sqrt{2}}\begin{pmatrix} n \\ e^{i\varphi_k} \end{pmatrix}.$$

Here $n = \pm 1$ denotes the band number, and $k_x + ik_y = ke^{i\varphi_k}$. In real space, these eigenmodes take the form $\psi_{n,\mathbf{k}} = \frac{1}{\sqrt{2A}}\begin{pmatrix} n \\ e^{i\varphi_k} \end{pmatrix} e^{i\mathbf{k}\cdot\mathbf{r}}$, where A is the area of the lattice to keep track of the normalization. The eigenstates of the pseudospin (Pauli matrix σ_z) are given by:

$$\frac{\sigma_z}{2}\chi_{S,s} = s\chi_{S,s}, \chi_{\frac{1}{2},\frac{1}{2}} = \begin{pmatrix} 1 \\ 0 \end{pmatrix}, \chi_{\frac{1}{2},-\frac{1}{2}} = \begin{pmatrix} 0 \\ 1 \end{pmatrix}.$$

The beam excites initially only one value of the pseudospin. Because the HCL is diagonal in the sublattice basis, this means that initially we excite only one of the two sublattices. The complex amplitude of the electric field of the initial excitation is $\psi_{l,s}(r, \varphi_r, z=0) = \psi_0 r^l e^{il\varphi_r} \exp(-r^2/a_0^2)\,\chi_{S,s}$. We can rewrite it in momentum space as follows (since we study linear dynamics, ψ_0 can be rescaled without affecting the results):

$$\psi_{l,s}(r, \varphi_r, z=0) = \psi_0 \int d^2k \frac{1}{\sqrt{A}}\chi_{S,s} f(\mathbf{k}) e^{i\mathbf{k}\cdot\mathbf{r}},$$

here function $f(\mathbf{k})$ depends on the transverse profile and the phase structure of the initial excitation, and the integral is taken over the whole k-space. For our input beam

$$f(\mathbf{k}) = (k_x + ik_y)^l \exp\left(-\frac{k_x^2+k_y^2}{k_F^2}\right) = k^l e^{il\varphi_k} \exp\left(-\frac{k^2}{k_F^2}\right),$$

where k_F defines the width of the beam in k-space. For a given l, there are two possible initial excitations, $\psi_{l,s=\frac{1}{2}}$ or $\psi_{l,s=-\frac{1}{2}}$, which can be written via superposition of the eigenmodes of the HCL as follows:

$$\psi_{l,s=\frac{1}{2}} = \sum_n \int d^2k \left\langle \psi_{n,\mathbf{k}} \middle| \psi_{l,s=\frac{1}{2}} \right\rangle \psi_{n,\mathbf{k}},$$

and

$$\psi_{l,s=-\frac{1}{2}} = \sum_n \int d^2k \left\langle \psi_{n,\mathbf{k}} \middle| \psi_{l,s=-\frac{1}{2}} \right\rangle \psi_{n,\mathbf{k}}.$$

The scalar products of the initial wavepacket with the eigenmodes are easily evaluated:

$$\left\langle \psi_{n,\mathbf{k}} \middle| \psi_{l,s=\frac{1}{2}} \right\rangle = \psi_0 \frac{n}{\sqrt{2}} f(\mathbf{k}),$$

and

$$\left\langle \psi_{n,\mathbf{k}} \middle| \psi_{l,s=-\frac{1}{2}} \right\rangle = \psi_0 \frac{e^{-i\varphi_k}}{\sqrt{2}} f(\mathbf{k}).$$

The initial states are then

$$\psi_{l,s=\frac{1}{2}} = \psi_0 \sum_n \int d^2k \frac{1}{2\sqrt{A}} \begin{pmatrix} 1 \\ ne^{i\varphi_k} \end{pmatrix} e^{i\mathbf{k}\cdot\mathbf{r}} f(\mathbf{k}),$$

and

$$\psi_{l,s=-\frac{1}{2}} = \psi_0 \sum_n \int d^2k \frac{1}{2\sqrt{A}} \begin{pmatrix} ne^{-i\varphi_k} \\ 1 \end{pmatrix} e^{i\mathbf{k}\cdot\mathbf{r}} f(\mathbf{k}).$$

The eigenmodes in the HCL evolve dynamically, each one with its own propagation constant:

$$\psi_{l,s=\frac{1}{2}} = \psi_0 \sum_n \int d^2k \frac{1}{2\sqrt{A}} \begin{pmatrix} 1 \\ ne^{i\varphi_k} \end{pmatrix} e^{i\mathbf{k}\cdot\mathbf{r} - in\beta_0 kz} f(\mathbf{k}),$$

and

$$\psi_{l,s=-\frac{1}{2}} = \psi_0 \sum_n \int d^2k \frac{1}{2\sqrt{A}} \begin{pmatrix} ne^{-i\varphi_k} \\ 1 \end{pmatrix} e^{i\mathbf{k}\cdot\mathbf{r} - in\beta_0 kz} f(\mathbf{k}),$$

where $\beta_0 = \kappa/2$. After sufficiently long time of propagation, we have far field dynamics as show below.

Let us now assume that we have excited pseudospin $s = 1/2$. The evolving complex amplitude of the electric field is given by:

$$\psi_{l,s=\frac{1}{2}}(r,\varphi_r,z) = \frac{\psi_0}{2\sqrt{A}}\sum_{n=\pm1}\int d^2k\, f(\mathbf{k})\chi_{\frac{1}{2},\frac{1}{2}}e^{i\mathbf{k}\cdot\mathbf{r}-in\beta_0 kz} + \frac{\psi_0}{2\sqrt{A}}\sum_{n=\pm1}\int d^2k\, ne^{i\varphi_k}f(\mathbf{k})\chi_{\frac{1}{2},-\frac{1}{2}}e^{i\mathbf{k}\cdot\mathbf{r}-in\beta_0 kz}.$$

After taking the sum over the band index n, we get:

$$\psi_{l,s=\frac{1}{2}}(r,\varphi_r,z) = \frac{\psi_0}{\sqrt{A}}\int_0^{2\pi}\int_0^{\infty}\mathrm{d}\varphi_k\, k\mathrm{d}k\, f(\mathbf{k})\chi_{\frac{1}{2},\frac{1}{2}}e^{i\mathbf{k}\cdot\mathbf{r}}\cos(\beta_0 kz) + \frac{-\psi_0}{\sqrt{A}}\int_0^{2\pi}\int_0^{\infty}\mathrm{d}\varphi_k\, k\mathrm{d}k\, f(\mathbf{k})e^{i\varphi_k}\chi_{\frac{1}{2},-\frac{1}{2}}e^{i\mathbf{k}\cdot\mathbf{r}}i\sin(\beta_0 kz).$$

After substituting $f(\mathbf{k})$ and $\mathbf{k}\cdot\mathbf{r} = kr\cos(\varphi_k - \varphi_r)$, we get:

$$\psi_{l,s=\frac{1}{2}}(r,\varphi_r,z) = \frac{\psi_0}{\sqrt{A}}\chi_{\frac{1}{2},\frac{1}{2}}\int_0^{2\pi}\int_0^{\infty}\mathrm{d}\varphi_k\, k\mathrm{d}k\, k^l e^{il\varphi_k}e^{-\frac{k^2}{k_F^2}}e^{ikr\cos(\varphi_k-\varphi_r)}\cos(\beta_0 kz) + \frac{-\psi_0}{\sqrt{A}}\chi_{\frac{1}{2},-\frac{1}{2}}\int_0^{2\pi}\int_0^{\infty}\mathrm{d}\varphi_k\, k\mathrm{d}k\, k^l e^{il\varphi_k}e^{-\frac{k^2}{k_F^2}}e^{i\varphi_k}e^{ikr\cos(\varphi_k-\varphi_r)}i\sin(\beta_0 kz).$$

We now change the variable of integration from φ_k to $\varphi = \varphi_k - \varphi_r$ to get:

$$\psi_{l,s=\frac{1}{2}} = \frac{\psi_0}{\sqrt{A}}e^{il\varphi_r}\chi_{\frac{1}{2},\frac{1}{2}}\int_0^{2\pi}\int_0^{\infty}\mathrm{d}\varphi\, k\mathrm{d}k\, k^l e^{il\varphi}e^{-k^2/k_F{}^2}e^{ikr\cos(\varphi)}\cos(\beta_0 kz) + \frac{-\psi_0}{\sqrt{A}}e^{i(l+1)\varphi_r}\chi_{\frac{1}{2},-\frac{1}{2}}\int_0^{2\pi}\int_0^{\infty}\mathrm{d}\varphi\, k\mathrm{d}k\, k^l e^{i(l+1)\varphi}e^{-k^2/k_F{}^2}e^{ikr\cos(\varphi)}i\sin(\beta_0 kz).$$

To clarify the mathematical structure of the output, we introduce the g-functions defined by,

$$g_{\frac{1}{2},\frac{1}{2}}(r,z) = \frac{\psi_0}{\sqrt{A}}\int_0^{2\pi}\int_0^{\infty}\mathrm{d}\varphi\, k\mathrm{d}k\, k^l e^{il\varphi}e^{-k^2/k_F{}^2}e^{ikr\cos(\varphi)}\cos(\beta_0 kz),$$
$$g_{\frac{1}{2},-\frac{1}{2}}(r,z) = \frac{-\psi_0}{\sqrt{A}}\int_0^{2\pi}\int_0^{\infty}\mathrm{d}\varphi\, k\mathrm{d}k\, k^l e^{i(l+1)\varphi}e^{-k^2/k_F{}^2}e^{ikr\cos(\varphi)}i\sin(\beta_0 kz),$$

which contain the radial and z-dependence of the optical field. This gives us the final result:

$$\psi_{l,s=\frac{1}{2}}(\mathbf{r},z) = e^{il\varphi_r}\chi_{\frac{1}{2},\frac{1}{2}}g_{\frac{1}{2},\frac{1}{2}}(r,z) + e^{i(l+1)\varphi_r}\chi_{\frac{1}{2},-\frac{1}{2}}g_{\frac{1}{2},-\frac{1}{2}}(r,z),$$

which is exactly the result presented in Eq. (5) in main text. The g-functions are illustrated in Supplementary Figures 3. The vorticity of the initially unexcited pseudospin component -1/2 underwent topological charge conversion $l \rightarrow l + 2s$. Fully analogous calculation explains dynamics for the other initial excitations in the HCL.

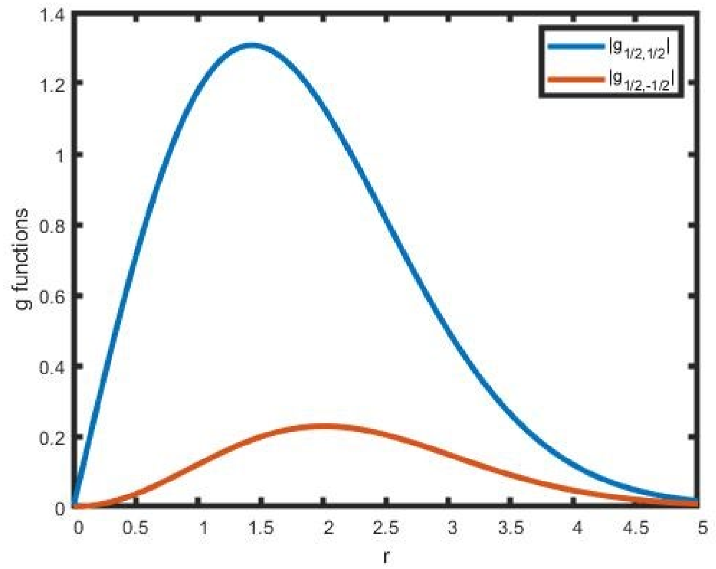

Supplementary Figure 3. The g-functions for the honeycomb lattice. We clearly see the total beam intensity at the output has a donut shaped structure as expected for conical diffraction. For the illustration, the parameters ψ_0, A, k_F, κ were taken as unity, and $z = 0.2$.

Lieb lattice – dynamics via expansion over the eigenstates: In a fully equivalent manner, we can describe dynamics in the Lieb lattices. The Hamiltonian describing conical intersection of the Lieb lattice is

$$H = \kappa\left(S_x k_x + S_y k_y\right), \tag{3}$$

where the $S_{x,y,z}$ matrices represent pseudospin 1:

$$S_x = \begin{pmatrix} 0 & 1 & 0 \\ 1 & 0 & 0 \\ 0 & 0 & 0 \end{pmatrix}, S_y = \begin{pmatrix} 0 & 0 & 0 \\ 0 & 0 & 1 \\ 0 & 1 & 0 \end{pmatrix}, S_z = \begin{pmatrix} 0 & 0 & -i \\ 0 & 0 & 0 \\ i & 0 & 0 \end{pmatrix}.$$

In this case, the propagation constants and the eigenmodes of the Hamiltonian are:

$$\beta_{n,\mathbf{k}} = n\kappa\sqrt{{k_x}^2 + {k_y}^2} = n\kappa k, n = -1,0,1,$$

$$\psi_{0,\mathbf{k}} = \frac{1}{k}\begin{pmatrix} -k_y \\ 0 \\ k_x \end{pmatrix}, \text{ for } \beta_{0,\mathbf{k}} = 0, \tag{4}$$

$$\psi_{\pm 1,\mathbf{k}} = \frac{1}{k\sqrt{2}}\begin{pmatrix} k_x \\ \pm k \\ k_y \end{pmatrix}, \text{ for } \beta_{\pm 1,\mathbf{k}} = \pm\kappa k. \tag{5}$$

Here again $k_x + ik_y = ke^{i\varphi_k}$. The eigenstates of the pseudospin matrix S_z are given by $S_z\chi_{S,s} = s\chi_{S,s}$:

$$\chi_{1,0} = \begin{pmatrix} 0 \\ 1 \\ 0 \end{pmatrix}, \chi_{1,1} = \frac{1}{\sqrt{2}} \begin{pmatrix} -i \\ 0 \\ 1 \end{pmatrix}, \chi_{1,-1} = \frac{1}{\sqrt{2}} \begin{pmatrix} i \\ 0 \\ 1 \end{pmatrix}.$$

The initial excitation is described by

$$\psi_{l,s}(r, \varphi_r, z = 0) = \psi_0 \int d^2k \frac{1}{\sqrt{A}} \chi_{S,s} f(\mathbf{k}) e^{i\mathbf{k}\cdot\mathbf{r}}.$$

For a given l, there are three possible initial conditions: $\psi_{l,s=1}$, $\psi_{l,s=0}$, and $\psi_{l,s=-1}$. Let us write the first of these initial conditions as a superposition of the Lieb lattice eigenstates (the calculation is fully equivalent for the other initial conditions):

$$\psi_{l,s=1} = \sum_n \int d^2k \left\langle \psi_{n,\mathbf{k}} \middle| \psi_{l,s=1} \right\rangle \psi_{n,\mathbf{k}}.$$

The scalar products of the initial wavepacket with the eigenmodes are easily found to be (the eigenmodes in Supplementary Equations 4 and 5are for this purpose used in real space as for the HCL above)

$$\left\langle \psi_{0,\mathbf{k}} \middle| \psi_{l,s=1} \right\rangle = \psi_0 \frac{1}{\sqrt{2}} f(\mathbf{k}) e^{i\varphi_k}, \text{ and } \left\langle \psi_{\pm,\mathbf{k}} \middle| \psi_{l,s=1} \right\rangle = \psi_0 \frac{-i}{2} f(\mathbf{k}) e^{i\varphi_k}.$$

For this initial state we now have

$$\psi_{l,s=1}(r, \varphi_r, z = 0) = \int d^2k \left(\psi_0 \frac{1}{\sqrt{2}} f(\mathbf{k}) e^{i\varphi_k}\right) \frac{1}{\sqrt{A}} \frac{1}{k} \begin{pmatrix} -k_y \\ 0 \\ k_x \end{pmatrix} e^{i\mathbf{k}\cdot\mathbf{r}} +$$

$$\sum_{n=\pm 1} \int d^2k \left(\psi_0 \frac{-i}{2} f(\mathbf{k}) e^{i\varphi_k}\right) \frac{1}{\sqrt{A}} \frac{1}{k\sqrt{2}} \begin{pmatrix} k_x \\ nk \\ k_y \end{pmatrix} e^{i\mathbf{k}\cdot\mathbf{r}},$$

The eigenmodes evolve dynamically:

$$\psi_{l,s=1}(r, \varphi_r, z) = \int d^2k \left(\psi_0 \frac{1}{\sqrt{2}} f(\mathbf{k}) e^{i\varphi_k}\right) \frac{1}{\sqrt{A}} \frac{1}{k} \begin{pmatrix} -k_y \\ 0 \\ k_x \end{pmatrix} e^{i\mathbf{k}\cdot\mathbf{r}} +$$

$$\sum_{n=\pm 1} \int d^2k \left(\psi_0 \frac{-i}{2} f(\mathbf{k}) e^{i\varphi_k}\right) \frac{1}{\sqrt{A}} \frac{1}{k\sqrt{2}} \begin{pmatrix} k_x \\ nk \\ k_y \end{pmatrix} e^{i\mathbf{k}\cdot\mathbf{r} - in\kappa kz},$$

By using $\frac{k_y}{k} = \frac{e^{i\varphi_k} - e^{-i\varphi_k}}{2i}$ and $\frac{k_x}{k} = \frac{e^{i\varphi_k} + e^{-i\varphi_k}}{2}$, we obtain

$$\psi_{l,s=1}(r,\varphi_r,z)=\int d^2k\ \left(\psi_0\frac{1}{\sqrt{2}}f(\mathbf{k})e^{i\varphi_k}\right)\frac{1}{\sqrt{A}}\begin{pmatrix}-\frac{e^{i\varphi_k}-e^{-i\varphi_k}}{2i}\\ 0\\ \frac{e^{i\varphi_k}+e^{-i\varphi_k}}{2}\end{pmatrix}e^{i\mathbf{k}\cdot\mathbf{r}}+$$

$$\sum_{n=\pm1}\int d^2k\ \left(\psi_0\frac{-i}{2}f(\mathbf{k})e^{i\varphi_k}\right)\frac{1}{\sqrt{A}}\frac{1}{\sqrt{2}}\begin{pmatrix}\frac{e^{i\varphi_k}+e^{-i\varphi_k}}{2}\\ n\\ \frac{e^{i\varphi_k}-e^{-i\varphi_k}}{2i}\end{pmatrix}e^{i\mathbf{k}\cdot\mathbf{r}-in\kappa kz},$$

Next, the column matrices are expressed in terms of the pseudospin eigenstates:

$$\begin{pmatrix}-\frac{e^{i\varphi_k}-e^{-i\varphi_k}}{2i}\\ 0\\ \frac{e^{i\varphi_k}+e^{-i\varphi_k}}{2}\end{pmatrix}=\frac{e^{i\varphi_k}}{2}\begin{pmatrix}i\\0\\1\end{pmatrix}+\frac{e^{-i\varphi_k}}{2}\begin{pmatrix}-i\\0\\1\end{pmatrix}=\frac{e^{i\varphi_k}}{\sqrt{2}}\chi_{1,-1}+\frac{e^{-i\varphi_k}}{\sqrt{2}}\chi_{1,1},$$

$$\begin{pmatrix}\frac{e^{i\varphi_k}+e^{-i\varphi_k}}{2}\\ n\\ \frac{e^{i\varphi_k}-e^{-i\varphi_k}}{2i}\end{pmatrix}=\begin{pmatrix}0\\n\\0\end{pmatrix}+\frac{e^{i\varphi_k}}{2i}\begin{pmatrix}i\\0\\1\end{pmatrix}-\frac{e^{-i\varphi_k}}{2i}\begin{pmatrix}-i\\0\\1\end{pmatrix}=n\chi_{1,0}+\frac{e^{i\varphi_k}}{\sqrt{2}i}\chi_{1,-1}-\frac{e^{-i\varphi_k}}{\sqrt{2}i}\chi_{1,1}.$$

We collect the terms according to the pseudospin:

$$\psi_{l,s=1}(r,\varphi_r,z)=\int d^2k\left(\left(\psi_0\frac{-i}{2}f(\mathbf{k})e^{i\varphi_k}\right)\left(\frac{1}{\sqrt{A}}\frac{1}{\sqrt{2}}e^{i\mathbf{k}\cdot\mathbf{r}}\right)2\ i\ (-1)\sin(\kappa kz)\right)\chi_{1,0}+$$
$$\int d^2k\left(\left(\psi_0\frac{1}{\sqrt{2}}f(\mathbf{k})e^{i\varphi_k}\right)\left(\frac{1}{\sqrt{A}}e^{i\mathbf{k}\cdot\mathbf{r}}\right)\frac{e^{i\varphi_k}}{\sqrt{2}}+\right.$$
$$\left.\left(\psi_0\frac{-i}{2}f(\mathbf{k})e^{i\varphi_k}\right)\left(\frac{1}{\sqrt{A}}\frac{1}{\sqrt{2}}e^{i\mathbf{k}\cdot\mathbf{r}}\right)\frac{e^{i\varphi_k}}{\sqrt{2}i}2\cos(\kappa kz)\right)\chi_{1,-1}+$$
$$\int d^2k\left(\left(\psi_0\frac{1}{\sqrt{2}}f(\mathbf{k})e^{i\varphi_k}\right)\left(\frac{1}{\sqrt{A}}e^{i\mathbf{k}\cdot\mathbf{r}}\right)\frac{e^{-i\varphi_k}}{\sqrt{2}}-\left(\psi_0\frac{-i}{2}f(\mathbf{k})e^{i\varphi_k}\right)\left(\frac{1}{\sqrt{A}}\frac{1}{\sqrt{2}}e^{i\mathbf{k}\cdot\mathbf{r}}\right)\frac{e^{-i\varphi_k}}{\sqrt{2}i}2\cos(\kappa kz)\right)\chi_{1,1}.$$

We can use $1-\cos(2x)=2\sin^2(x)$ and $1+\cos(2x)=2\cos^2(x)$ to get:

$$\psi_{l,s=1}(r,\varphi_r,z)=\frac{\psi_0}{\sqrt{A}}\int_0^{2\pi}\int_0^{\infty}\mathrm{d}\varphi_k\ k\mathrm{d}k\ f(\mathbf{k})\chi_{1,1}e^{i\mathbf{k}\cdot\mathbf{r}}\cos^2\left(\frac{\kappa kz}{2}\right)+$$
$$\frac{\psi_0}{\sqrt{A}}\int_0^{2\pi}\int_0^{\infty}\mathrm{d}\varphi_k\ k\mathrm{d}k\ f(\mathbf{k})e^{2i\varphi_k}\chi_{1,-1}e^{i\mathbf{k}\cdot\mathbf{r}}\sin^2(\frac{\kappa kz}{2})+$$
$$\frac{-\psi_0}{\sqrt{2A}}\int_0^{2\pi}\int_0^{\infty}\mathrm{d}\varphi_k\ k\mathrm{d}k\ f(\mathbf{k})e^{i\varphi_k}\chi_{1,0}e^{i\mathbf{k}\cdot\mathbf{r}}\sin(\kappa kz).$$

By substituting the expression for $f(\mathbf{k})$, $\mathbf{k}\cdot\mathbf{r}=kr\cos(\varphi_k-\varphi_r)$, and performing a change of variable of integration from φ_k to $\varphi=\varphi_k-\varphi_r$ as we did for the HCL, it is straightforward to get

$$\psi_{l,s=1} = e^{il\varphi_r}\chi_{1,1}\frac{\psi_0}{\sqrt{A}}\int_0^{2\pi}\int_0^{\infty} \mathrm{d}\varphi\, k\mathrm{d}k\, k^l e^{il\varphi} e^{-k^2/k_F{}^2} e^{ikr\cos(\varphi)}\cos^2\left(\frac{\kappa kz}{2}\right) +$$
$$e^{i(l+2)\varphi_r}\chi_{1,-1}\frac{\psi_0}{\sqrt{A}}\int_0^{2\pi}\int_0^{\infty} \mathrm{d}\varphi\, k\mathrm{d}k\, k^l e^{i(l+2)\varphi} e^{-k^2/k_F{}^2} e^{ikr\cos(\varphi)}\sin^2\left(\frac{\kappa kz}{2}\right) +$$
$$e^{i(l+1)\varphi_r}\chi_{1,0}\frac{-\psi_0}{\sqrt{2A}}\int_0^{2\pi}\int_0^{\infty} \mathrm{d}\varphi\, k\mathrm{d}k\, k^l e^{i(l+1)\varphi} e^{-k^2/k_F{}^2} e^{ikr\cos(\varphi)}\sin(\kappa kz).$$

This is exactly Eq. (6) in the main text, with the g-functions defined as

$$g_{1,1}(r,z) = \frac{\psi_0}{\sqrt{A}}\int_0^{2\pi}\int_0^{\infty} \mathrm{d}\varphi\, k\mathrm{d}k\, k^l e^{il\varphi} e^{-\frac{k^2}{k_F{}^2}} e^{ikr\cos(\varphi)}\cos^2\left(\frac{\kappa kz}{2}\right),$$

$$g_{1,0}(r,z) = \frac{-\psi_0}{\sqrt{2A}}\int_0^{2\pi}\int_0^{\infty} \mathrm{d}\varphi\, k\mathrm{d}k\, k^l e^{i(l+1)\varphi} e^{-\frac{k^2}{k_F{}^2}} e^{ikr\cos(\varphi)}\sin(\kappa kz)\,,$$

$$g_{1,-1}(r,z) = \frac{\psi_0}{\sqrt{A}}\int_0^{2\pi}\int_0^{\infty} \mathrm{d}\varphi\, k\mathrm{d}k\, k^l e^{i(l+2)\varphi} e^{-\frac{k^2}{k_F{}^2}} e^{ikr\cos(\varphi)}\sin^2\left(\frac{\kappa kz}{2}\right).$$

The g-functions are illustrated in Supplementary Figure 4. The OAM of the initially excited component remains unchanged, while the OAM in the initially unexcited components follow the rule $l + s = l' + s'$.

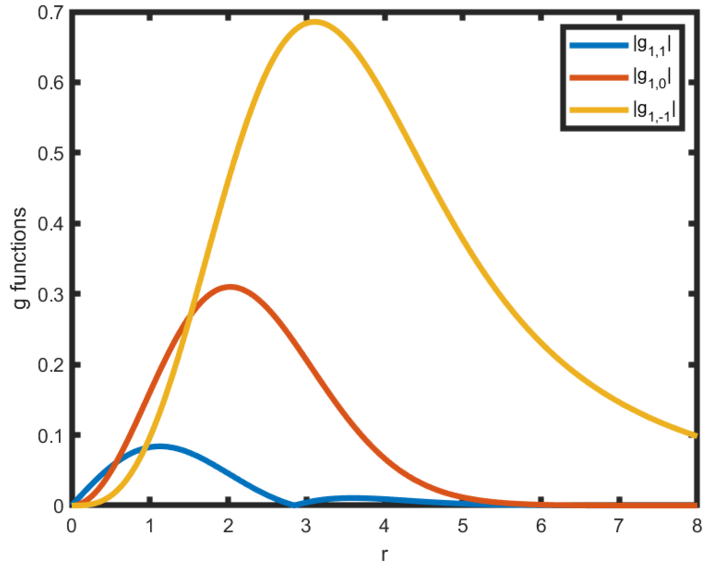

Supplementary Figure 4. The g-functions for the Lieb lattice. We see that for the Lieb lattice, as well as for the HCL, the total beam intensity at the output has a donut shaped structure. For the illustration, the parameters ψ_0, A, k_F, κ were taken as unity, and $z = 0.2$.

Weyl Hamiltonian – expansion over the eigenstates: In 3D, the Weyl Hamiltonian is given by

$H_{\text{Weyl}} = \frac{\kappa}{2}\left(\sigma_x k_x + \sigma_y k_y + \sigma_z k_z\right)$.

The eigenvalues and eigenstates of H_{Weyl} are given by

$$E_{n,\mathbf{k}} = n\frac{\kappa}{2}\sqrt{{k_x}^2 + {k_y}^2 + {k_z}^2}, n = \pm 1,$$

$$\psi_{+1,\mathbf{k}} = \begin{pmatrix} \cos\left(\frac{\theta_k}{2}\right) e^{-i\varphi_k} \\ \sin\left(\frac{\theta_k}{2}\right) \end{pmatrix},$$

$$\psi_{-1,\mathbf{k}} = \begin{pmatrix} \sin\left(\frac{\theta_k}{2}\right) e^{-i\varphi_k} \\ -\cos\left(\frac{\theta_k}{2}\right) \end{pmatrix},$$

where we have used spherical coordinates (k, θ_k, φ_k). The pseudospin eigenstates are identical as for the HCL. Suppose that initially we excite the modes around the Weyl point with a rotationally symmetric Gaussian-like distribution, $f(\mathbf{k}) = f(k)$, in an arbitrary pseudospin state,

$$\psi_{0,\alpha\beta}(\mathbf{r}, t = 0) = \psi_0 \int d^3k \frac{1}{\sqrt{V}} \binom{\alpha}{\beta} f(k) e^{i\mathbf{k}\cdot\mathbf{r}}.$$

Here V denotes the volume of the system used to normalize the eigenstates. Next, we write the initial conditions as a superposition of the Weyl eigenstates:

$$\psi_{0,\alpha\beta}(\mathbf{r}, t = 0) = \sum_n \int d^3k \left\langle \psi_{n,\mathbf{k}} \middle| \psi_{0,\alpha\beta} \right\rangle \psi_{n,\mathbf{k}}.$$

Here, the scalar products of the initial wavepacket with the eigenmodes are evaluated to be

$$\left\langle \psi_{+1,\mathbf{k}} \middle| \psi_{0,\alpha\beta} \right\rangle = \psi_0 f(\mathbf{k}) \left(\alpha \cos\left(\frac{\theta_k}{2}\right) e^{i\varphi_k} + \beta \sin\left(\frac{\theta_k}{2}\right)\right),$$

and

$$\left\langle \psi_{-1,\mathbf{k}} \middle| \psi_{0,\alpha\beta} \right\rangle = \psi_0 f(\mathbf{k}) \left(\alpha \sin\left(\frac{\theta_k}{2}\right) e^{i\varphi_k} - \beta \cos\left(\frac{\theta_k}{2}\right)\right).$$

Subsequent evolution from this initial state is given by:

$$\psi_{0,\alpha\beta}(\mathbf{r}, t) = \psi_0 \int d^3k \frac{1}{\sqrt{V}} e^{i\mathbf{k}\cdot\mathbf{r}} f(k) \left[\left(\alpha \cos\left(\frac{\theta_k}{2}\right) e^{i\varphi_k} + \beta \sin\left(\frac{\theta_k}{2}\right)\right) \begin{pmatrix} \cos\left(\frac{\theta_k}{2}\right) e^{-i\varphi_k} \\ \sin\left(\frac{\theta_k}{2}\right) \end{pmatrix} e^{i\omega_0 t} + \left(\alpha \sin\left(\frac{\theta_k}{2}\right) e^{i\varphi_k} - \beta \cos\left(\frac{\theta_k}{2}\right)\right) \begin{pmatrix} \sin\left(\frac{\theta_k}{2}\right) e^{-i\varphi_k} \\ -\cos\left(\frac{\theta_k}{2}\right) \end{pmatrix} e^{-i\omega_0 t}\right],$$

where $\omega_0 = \frac{\kappa}{2}k$. For concreteness, we assume from this point on that we have excited the pseudospin component $\chi_{\frac{1}{2},\frac{1}{2}} = \begin{pmatrix}1\\0\end{pmatrix}$, i.e., if $\alpha = 1, \beta = 0$

$$\psi_{0,\frac{1}{2}}(\mathbf{r},t) = \psi_0 \int d^3k\ \frac{1}{\sqrt{V}} e^{i\mathbf{k}\cdot\mathbf{r}} f(k) \left[\cos\left(\frac{\theta_k}{2}\right) e^{i\varphi_k} \begin{pmatrix}\cos\left(\frac{\theta_k}{2}\right) e^{-i\varphi_k}\\ \sin\left(\frac{\theta_k}{2}\right)\end{pmatrix} e^{i\omega_0 t} + \sin\left(\frac{\theta_k}{2}\right) e^{i\varphi_k} \begin{pmatrix}\sin\left(\frac{\theta_k}{2}\right) e^{-i\varphi_k}\\ -\cos\left(\frac{\theta_k}{2}\right)\end{pmatrix} e^{-i\omega_0 t}\right],$$

Now we collect the terms corresponding to the two pseudospin components

$$\psi_{0,\frac{1}{2}}(\mathbf{r},t) = \psi_0 \int d^3k\ \frac{1}{\sqrt{V}} e^{i\mathbf{k}\cdot\mathbf{r}} f(k) \left\{\begin{pmatrix}0\\1\end{pmatrix} \sin(\theta_k)\ \ e^{i\varphi_k}\, i\ \sin(\omega_0 t) + \begin{pmatrix}1\\0\end{pmatrix}\left[e^{i\omega_0 t}\cos^2\left(\frac{\theta_k}{2}\right) + e^{-i\omega_0 t}\sin^2\left(\frac{\theta_k}{2}\right)\right]\right\}.$$

If we assume that the initial excitation is a Gaussian function $f(k)$= $\exp\left(-\frac{k^2}{k_F^2}\right)$, we have

$$\psi_{0,\frac{1}{2}}(\mathbf{r},t) = \psi_0 \int d^3k\ \frac{1}{\sqrt{V}} e^{i\mathbf{k}\cdot\mathbf{r}} \exp\left(-\frac{k^2}{k_F^2}\right)\left\{\sin(\theta_k)\begin{pmatrix}0\\1\end{pmatrix}\ \ e^{i\varphi_k}\, i\ \sin(\omega_0 t) + \begin{pmatrix}1\\0\end{pmatrix}\left[e^{i\omega_0 t}\cos^2\left(\frac{\theta_k}{2}\right) + e^{-i\omega_0 t}\sin^2\left(\frac{\theta_k}{2}\right)\right]\right\}.$$

By substituting the expression for $\mathbf{k}\cdot\mathbf{r} = kr\sin\theta_k\sin\theta_r\cos(\varphi_k - \varphi_r) + kr\cos\theta_k\cos\theta_r$, and performing a change for the azimuthal variable of integration from φ_k to $\varphi = \varphi_k - \varphi_r$ as we did for the HCL above, it is straightforward to get

$$\psi_{0,\frac{1}{2}}(r,\theta_r,\varphi_r,t) = \chi_{\frac{1}{2},\frac{1}{2}} g_{\frac{1}{2},\frac{1}{2}}(r,\theta_r,t) + e^{i\varphi_r}\chi_{\frac{1}{2},-\frac{1}{2}} g_{\frac{1}{2},-\frac{1}{2}}(r,\theta_r,t),$$

where

$$g_{\frac{1}{2},-\frac{1}{2}}(r,\theta_r,t) = \int_0^\infty dk \int_0^\pi k\sin\theta_k d\theta_k \int_0^{2\pi} kd\varphi \frac{\psi_0}{\sqrt{V}} e^{i(kr\sin\theta_k\sin\theta_r\cos\varphi + kr\cos\theta_k\cos\theta_r)} e^{-\frac{k^2}{k_F^2}} \sin\theta_k\ \ e^{i\varphi}\, i\sin(\omega_0 t),$$

and

$$g_{\frac{1}{2},\frac{1}{2}}(r,\theta_r,t) = \int_0^\infty dk \int_0^\pi k\sin\theta_k d\theta_k \int_0^{2\pi} kd\varphi \frac{\psi_0}{\sqrt{V}} e^{ikr(kr\sin\theta_k\sin\theta_r\cos\varphi + kr\cos\theta_k\cos\theta_r)} e^{-\frac{k^2}{k_F^2}} \left[e^{i\omega_0 t}\cos^2\left(\frac{\theta_k}{2}\right) + e^{-i\omega_0 t}\sin^2\left(\frac{\theta_k}{2}\right)\right].$$

Topological charge conversion for different band structures: In the main text, we argued that the observed mapping is fundamentally a topological phenomenon, which can thereby occur in other systems besides the conical intersections in the honeycomb and the Lieb lattices. Here we demonstrate the validity of our interpretation using two different band structures. The first one is the Hamiltonian

$$H_m = \begin{pmatrix} 0 & (k_x - ik_y)^m \\ (k_x + ik_y)^m & 0 \end{pmatrix} = k^m \begin{pmatrix} 0 & e^{-im\varphi_k} \\ e^{im\varphi_k} & 0 \end{pmatrix}.$$

The eigenstates of this Hamiltonian are easily found to be

$$\psi_{n,\mathbf{k}} = \frac{1}{\sqrt{2}}\begin{pmatrix} n \\ e^{im\varphi_k} \end{pmatrix},$$

where $n = \pm 1$ denotes the band number, and $k_x + ik_y = ke^{i\varphi_k}$, which yields $-i\oint \left\langle \psi_{n,\mathbf{k}} \middle| \frac{\partial}{\partial\varphi_k} \middle| \psi_{n,\mathbf{k}} \right\rangle d\varphi_k = m\pi$, so, the winding of the Berry phase is given here by $w = m$. If we excite this band structure with the optimal initial condition $\psi_0 \int d^2k \frac{1}{\sqrt{A}}\begin{pmatrix} 1 \\ 0 \end{pmatrix} f(\mathbf{k})e^{i\mathbf{k}\cdot\mathbf{r}}$, where $f(\mathbf{k}) = k^l e^{il\varphi_k}\exp\left(-\frac{k^2}{k_F^2}\right)$ and $l > 0$, the k-space vortex $e^{im\varphi_k}$ present in the unexcited component will be mapped into real space, yielding topological charge conversion $l \to l + w$, which supports the topological interpretation. The derivation is equivalent to the honeycomb case given above in Supplementary Discussion 2with $e^{i\varphi_k}$ replaced by $e^{im\varphi_k}$.

The second example is the Hamiltonian $H_0 = k\sigma_z$, where the eigenstates are spinors $\begin{pmatrix} 1 \\ 0 \end{pmatrix}$ and $\begin{pmatrix} 0 \\ 1 \end{pmatrix}$. In this case, we obtain $\oint \left\langle \psi_{n,\mathbf{k}} \middle| \frac{\partial}{\partial\varphi_k} \middle| \psi_{n,\mathbf{k}} \right\rangle d\varphi_k = 0$, and therefore $w = 0$. Because the Hamiltonian commutes with z −component of the pseudospin operator σ_z, by exciting the upper (lower) pseudospin, there will be no energy transfer to the other component during evolution, and therefore there will be no topological charge conversion, again consistent with $l \to l + w$.

Supplementary References:

1. Song, D. et al. Unveiling pseudospin and angular momentum in photonic graphene. *Nat. Commun.* **6** (2015).
2. Xia, S. et al. Demonstration of flat-band image transmission in optically induced Lieb photonic lattices. *Opt. Lett.* **41**, 1435-1438 (2016).
3. Peleg, O. et al. Conical diffraction and gap solitons in honeycomb photonic lattices, *Phys. Rev. Lett.* **98**, 103901 (2007).
4. Sepkhanov, R. A., Bazaliy, Y. B. & Beenakker, C. W. J. Extremal transmission at the Dirac point of a photonic band structure. *Phys. Rev. A* **75**, 063813 (2007).
5. Leykam, D., Bahat-Treidel, O. & Desyatnikov, A. S. Pseudospin and nonlinear conical diffraction in Lieb lattices. *Phys. Rev. A* **86**, 031805 (2012).
